# From Host-Microbiome Symbiosis to Clinical Translation: A Gut Microbiome Perspective on Radiation Enteritis

**DOI:** 10.7150/ijbs.134169

**Published:** 2026-07-20

**Authors:** Jieru Zhuang, Yun Zhong, Zhongyao Lin, Yongjun Lu, Hui Wang, Lingming Chen, Keli Yang, Delin Tan, Yunyi Qiu, Yuanxin Zhang, Huaiming Wang, Zhenhuang Ge

**Affiliations:** 1 Department of General Surgery (Colorectal surgery), The Sixth Affiliated Hospital, Sun Yat-sen University, Guangzhou, 510655, China.; 2 Guangdong Provincial Key Laboratory of Colorectal and Pelvic Floor Diseases, The Sixth Affiliated Hospital, Sun Yat-sen University, China.; 3 Key Laboratory of Human Microbiome and Chronic Diseases (Sun Yat-sen University), Ministry of Education, China.; 4 Biomedical Innovation Center, The Sixth Affiliated Hospital, Sun Yat-sen University, China.; 5 Run Ze Laboratory for Gastrointestinal Microbiome Study, School of Life Sciences, Sun Yat-sen University, Guangzhou, 510275, China.; 6 Institute of Laboratory Medicine, School of Medical Technology, Guangdong Medical University, Dongguan, 523808, China.; 7 Guangdong Provincial Key Laboratory of Diabetology, Guangzhou Key Laboratory of Mechanistic and Translational Obesity Research, The Third Affiliated Hospital of Sun Yat-sen University, Guangzhou, 510630, China.

**Keywords:** radiation enteritis, gut microbiota, microbial metabolites, host-microbiome interaction, gut-organ axis, microbiome-based therapy

## Abstract

Radiotherapy is an essential component of multimodal treatment for solid tumors, and more than half of patients with cancer receive radiation during their disease course. Because of the unique anatomical and physiological features of the intestine, radiation enteritis (RE) remains a common and clinically challenging complication of abdominal and pelvic irradiation, with limited effective treatment options. In this review, we re-examine RE from a host-microbiome perspective. We summarize classical pathophysiological mechanisms and discuss how radiotherapy reshapes gut microbial composition and metabolism. We also highlight the roles of microbial metabolites, including short-chain fatty acids, bile acids and tryptophan derivatives, in barrier repair, immune homeostasis and stem-cell regeneration. Finally, we discuss microbiome heterogeneity across disease phases, tumor types and host factors, as well as microbiota-mediated gut-brain, gut-cardiopulmonary, gut-skin and gut-bone-marrow axes involved in systemic radiation injury. We further outline microbiome-based strategies for individualized risk stratification and early prediction, and recent advances and limitations of probiotics and synbiotics, fecal microbiota transplantation, dietary and lifestyle interventions, drugs and natural products, engineered microbes and novel delivery systems, highlighting the gut microbiome as a promising entry point to improve prevention and treatment of RE and systemic radiation toxicity.

## Introduction

Radiotherapy is a cornerstone of cancer management and is widely deployed across a broad spectrum of malignancies worldwide. It is estimated that more than 50% of patients with cancer receive radiotherapy at some point during their treatment, whether with curative or palliative intent. Despite continued advances in precision radiotherapy, gastrointestinal injury remains a frequent and challenging complication in the treatment of abdominal and pelvic tumors, owing to the unique anatomical and physiological milieu of the bowel. Radiation enteritis (RE) affects up to 80% of patients undergoing pelvic or abdominal irradiation, leading to substantial deterioration in quality of life and, in some cases, necessitating treatment interruption or dose reduction, with potential compromise of oncological control.

RE is typically categorized into acute and chronic forms according to timing and pathophysiology. Acute RE emerges within weeks of initiating radiotherapy and is characterized by diarrhea, abdominal pain, nausea and tenesmus, reflecting direct epithelial injury and an acute inflammatory response. By contrast, chronic RE arises months to years after completion of treatment and is marked by intestinal fibrosis, endothelial proliferation and microvascular dysfunction, with clinical sequelae including persistent abdominal pain, altered bowel habits and intermittent bleeding^1^. Approximately 10-20% of patients with acute RE progress to a chronic phenotype; however, the mechanisms underpinning this transition remain incompletely defined.

The gut microbiome is increasingly implicated in the onset, progression and outcomes of RE. Radiotherapy profoundly alters microbial diversity and composition, typically with depletion of taxa traditionally associated with short-chain fatty acid (SCFA) production and barrier maintenance, such as members of Lachnospiraceae and the genus *Roseburia*, alongside expansion of potentially pathogenic or inflammation-associated lineages, including Enterobacteriaceae and Streptococcaceae^2^. Notably, these shifts are not merely consequences of radiation injury; they actively shape the host response to irradiation by influencing mucosal inflammation, epithelial barrier integrity and tissue repair^3^. On this basis, microbiome-directed interventions have become a salient target for the prevention and treatment of RE. Beyond local intestinal effects, the gut microbiome communicates with multiple host systems through the gut-brain, gut-cardiopulmonary, gut-skin and gut-bone-marrow axes. These interactions may contribute to the systemic effects of radiotherapy. This systems-level perspective broadens understanding of radiation-associated complications and may inform the development of integrated therapeutic strategies.

In this review, we synthesize current evidence on RE across clinical features, pathophysiology, microbiome alterations, therapeutic approaches and systemic effects, with particular emphasis on the central role of the gut microbiome and related translational advances. By integrating contemporary clinical practice with insights from microbiology and radiobiology, we aim to inform precision prevention, early diagnosis and individualized management of RE, and to outline future research priorities and challenges. To facilitate critical interpretation of this heterogeneous literature, we applied a tiered translational evidence framework throughout the review. Evidence was considered strong when clinical observations were accompanied by mechanistic validation in experimental models; moderate when clinical associations were biologically consistent with mechanisms demonstrated in independent preclinical studies; potentially translational when preclinical-only findings involved microorganisms, metabolites or pathways shared between humans and mice; and weak or exploratory when preclinical-only findings centered on mouse-specific taxa or mechanisms without clear human counterparts. This framework was used to distinguish clinically supported associations from mechanistic hypotheses and to highlight current limitations, inconsistencies and areas requiring prospective validation.

## 1. Classical pathophysiological mechanisms

Radiation-induced intestinal injury is a common complication among patients receiving abdominal or pelvic radiotherapy, arising from both direct and indirect effects of ionizing radiation on cells and tissues.

### 1.1 Radiation-induced oxidative injury and cell death

The direct effects of ionizing radiation primarily involve the ionization of DNA, resulting in single- and double-strand breaks and base damage. If unrepaired, these lesions activate cell-cycle checkpoints and apoptotic signaling pathways. Indirect effects occur via radiolysis of water, generating abundant reactive oxygen species (ROS), including hydroxyl radicals (•OH), superoxide anions (O_2_•^-^) and hydrogen peroxide (H_2_O_2_). These molecules oxidatively damage cellular constituents such as lipid membranes, proteins and nucleic acids. Excessive ROS also disrupt the mitochondrial electron transport chain, impair ATP synthesis and perturb energy metabolism, further amplifying ROS accumulation and creating a vicious cycle of oxidative injury^4^. Oxidative stress not only damages cellular architecture directly but also activates stress-responsive signaling, including ATM/ATR-p53 pathways and the NF-κB inflammatory cascade^5^.

Radiation-induced cell death is not monolithic; rather, it encompasses a network of death programs, including apoptosis, ferroptosis, necrosis and autophagy, which collectively drive tissue injury and organ dysfunction. Recent animal studies indicate a pivotal role for ferroptosis in radiation-induced intestinal damage. In Göttingen minipigs, ionizing radiation markedly reduced glutathione peroxidase 4 (GPX4) protein and GPX4 mRNA expression (by approximately 70-83% and 90-94%, respectively), a hallmark of ferroptosis^6^. Complementary mouse studies show that the ferroptosis inhibitor Ferrostatin-1 mitigates ROS generation, suppresses both ferroptosis and apoptosis, significantly improves survival after lethal irradiation and restores intestinal structural and functional integrity^7^.

**Figure 1:**
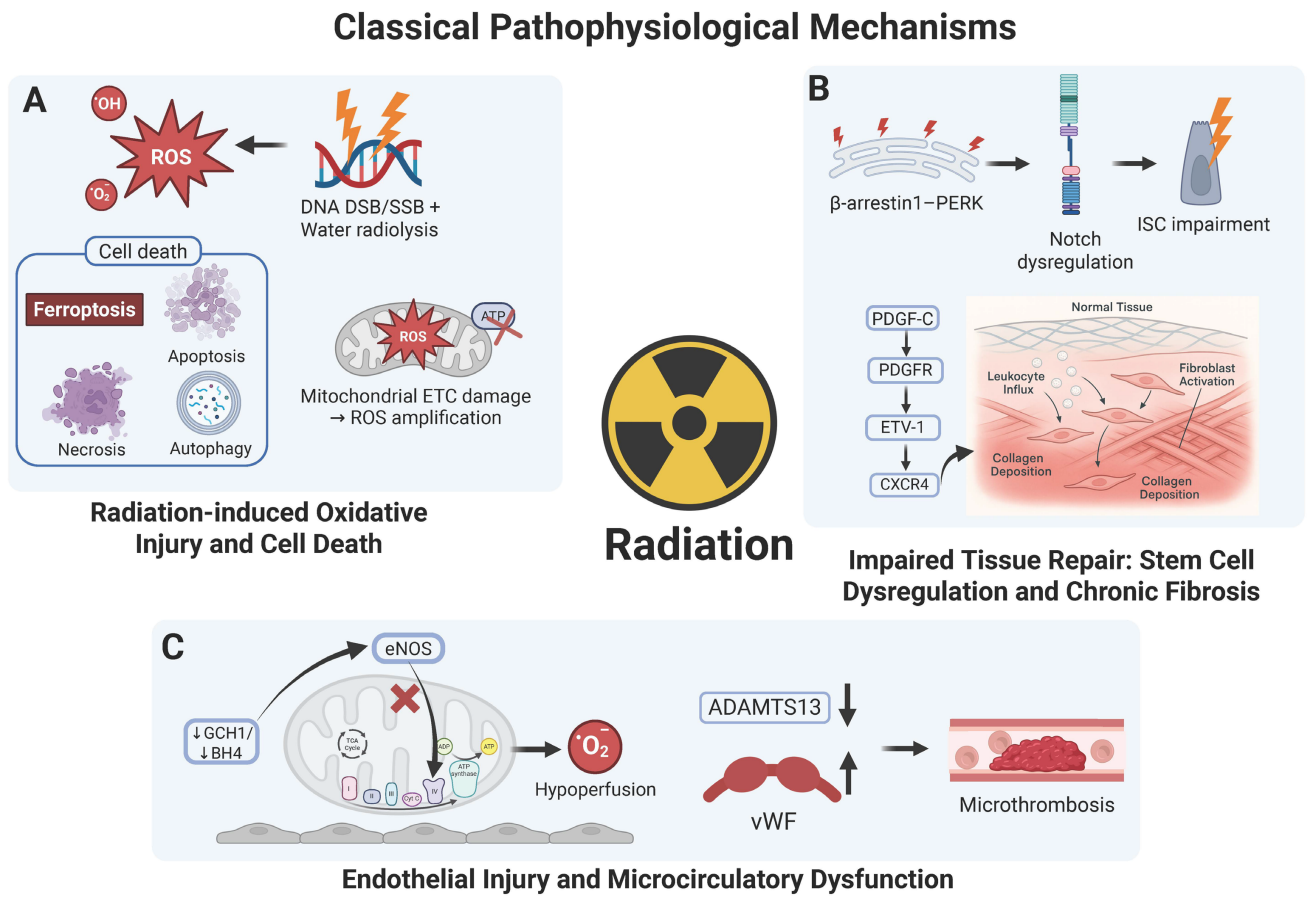
** Classical pathophysiological mechanisms of RE. (A)** Radiation-induced oxidative injury and cell death. **(B)** Impaired tissue repair associated with stem-cell dysregulation and chronic fibrosis. **(C)** Endothelial injury and microcirculatory dysfunction. ROS, reactive oxygen species; SSB/DSB, single-/double-strand breaks; ETC, electron transport chain; ATP, adenosine triphosphate; ISC, intestinal stem cell; TCA, tricarboxylic acid (cycle); ADAMTS13, a disintegrin and metalloproteinase with thrombospondin motifs 13; PERK, protein kinase R-like ER kinase; PDGF-C, platelet-derived growth factor-C; PDGFR, platelet-derived growth factor receptor; ETV1, ETS variant 1; CXCR4, C-X-C chemokine receptor 4; eNOS, endothelial nitric oxide synthase; GCH1, GTP cyclohydrolase 1; BH4, tetrahydrobiopterin; vWF, von Willebrand factor.

Notably, studies have revealed that microbes play a significant role in radiation-induced oxidative damage and cell death, as demonstrated by in vitro and murine research on a novel “theft-ferroptosis” mechanism. *Pseudomonas aeruginosa* secretes a bacterial 15-lipoxygenase that catalyzes host phosphatidylethanolamine to form 15-hydroperoxy-eicosatetraenoyl-phosphatidylethanolamine (15-HpETE-PE), while concomitantly releasing phospholipases that blunt endogenous anti-ferroptotic defenses. In mice, this synergy markedly exacerbates radiation-induced intestinal injury, shortening survival after total-body irradiation from 15 to 4 days^8^. Collectively, these data reveal complex microbiome-radiation interactions (Figure 1A).

### 1.2 Repair of impaired tissue: stem cell dysregulation and chronic fibrosis

The clinical burden of intestinal radiation injury stems not only from acute epithelial loss and barrier disruption, but also from chronic long-term repair damage. Two interlinked processes contribute to chronic disease: dysfunction of intestinal stem cells (ISCs) and a pathological inflammation-fibrosis cascade^9^.

Endoplasmic reticulum (ER) stress is a key mediator of radiation-induced stem cell dysfunction, constraining ISC proliferation and differentiation. In mouse models, β-arrestin-1 (βarr1) is markedly upregulated after irradiation. βarr1 directly binds and suppresses protein kinase R-like ER kinase (PERK)-mediated ER stress signaling, modulating downstream Notch activity and ultimately limiting ISC proliferative capacity and regenerative potential. In knockout mouse models, genetic ablation of β-arrestin 1 (βarr1) enhanced ISC proliferation and accelerated crypt regeneration. It also mitigated acute gastrointestinal syndrome and prolonged survival after lethal-dose irradiation^10^. When stem cell function is compromised and repair is delayed, unresolved acute inflammation evolves into chronic inflammation and fibrosis—the defining pathology of chronic RE. In mice, platelet-derived growth factor C (PDGF-C) engages PDGF receptors to activate an ETV1-driven CXCR4 signaling axis, sustaining immune-cell infiltration and fibroblast activation, with consequent collagen overdeposition and tissue fibrosis^11^. These mechanisms interlock to form a self-amplifying vicious cycle: impaired stem-cell function blunts epithelial regeneration and delays repair, while persistent inflammation and fibrogenesis erode the stem-cell niche—including vascular perfusion and stromal support—thereby deepening regenerative failure. This failed-regeneration-chronic inflammation-fibrosis axis may represent an important mechanism underlying the transition from acute to chronic RE (Figure 1B).

### 1.3 Endothelial injury and microcirculatory dysfunction

Endothelial damage is an early, prominent event in RE. Experimental studies show that ionizing radiation downregulates GTP cyclohydrolase 1 (Gch1), limiting tetrahydrobiopterin (BH4) synthesis and precipitating uncoupling of endothelial nitric oxide synthase (eNOS). Under physiological conditions, BH4 enables eNOS to generate nitric oxide (NO), which confers vasoprotection; when BH4 is deficient, eNOS instead produces superoxide, culminating in endothelial dysfunction and intestinal microischaemia. Clinically, patients exhibit a 45.9% reduction in plasma BH4 after radiotherapy, with intestinal perfusion falling to approximately 37% of baseline, indicating that endothelial dysfunction and microcirculatory compromise are key pathophysiological substrates of RE^12^.

Imbalance of the von Willebrand factor (vWF)/ADAMTS13 axis further aggravates vascular injury. Clinical observations indicate that radiotherapy increases prothrombotic vWF while relatively reducing its sole physiological cleaving protease, ADAMTS13, thereby tipping the hemostatic balance towards thrombosis. Using mice with platelet- and endothelium-specific deletion of vWF, vWF was shown to promote radiation-induced intestinal injury. Importantly, exogenous recombinant human ADAMTS13 restored vWF/ADAMTS13 equilibrium, attenuated inflammation and oxidative stress, and protected the intestine from radiation damage, nominating this axis as a potential biomarker and therapeutic target for radiation-induced gut injury^13^ (Figure 1C).

In summary, decades of work have elucidated canonical mechanisms of RE, encompassing direct cytotoxic effects, oxidative stress, multiple cell death programs, vascular injury, ER stress and an inflammation-fibrosis cascade. However, the translation of these established mechanisms into durable and effective therapies remains limited. Single-target interventions (for example, antioxidants, ferroptosis inhibitors, vasoprotective agents) show promise in animal models but have struggled to halt a disease driven by intertwined pathological processes in humans. Traditional paradigms also fail to explain the marked inter-individual variability under similar radiation regimens or predict why only 10-20% of acute cases progress to chronic disease, and these limitations are further compounded by the lack of reliable biomarkers. Moreover, a predominant focus on local intestinal injury has underappreciated systemic effects of radiotherapy and their bidirectional interplay with gut pathology. Crucially, the intestine has been viewed largely as a host organ, with insufficient consideration of the gut microbiome as a “hidden organ.” Emerging concepts, such as the “theft-ferroptosis” mechanism, show that microbes are not passive bystanders but active participants and modulators whose dysbiosis may link disparate pathological threads. These insights argue for a paradigm shift: from a host-centric view to a holistic host-microbiome interaction framework to explain individual variability, predict treatment responses, clarify acute-chronic transitions and enable rational, multi-target interventions.

## 2. Gut microbiome-associated regulatory network in the pathogenesis of RE

Limitations of single-target mechanistic studies underscore the need for a systems biology perspective. With advances in high-throughput sequencing, the gut microbiome has emerged as a potential modulator and biomarker of RE, linking environment, diet, immunity and metabolism. Microbiome-focused research offers a new framework to explain inter-individual variability, predict treatment responses and design personalized interventions. Crucially, the tractability of the microbiome and its metabolites provides actionable targets for clinical translation.

### 2.1 Impact of radiotherapy on gut microbiome composition

**Figure 2:**
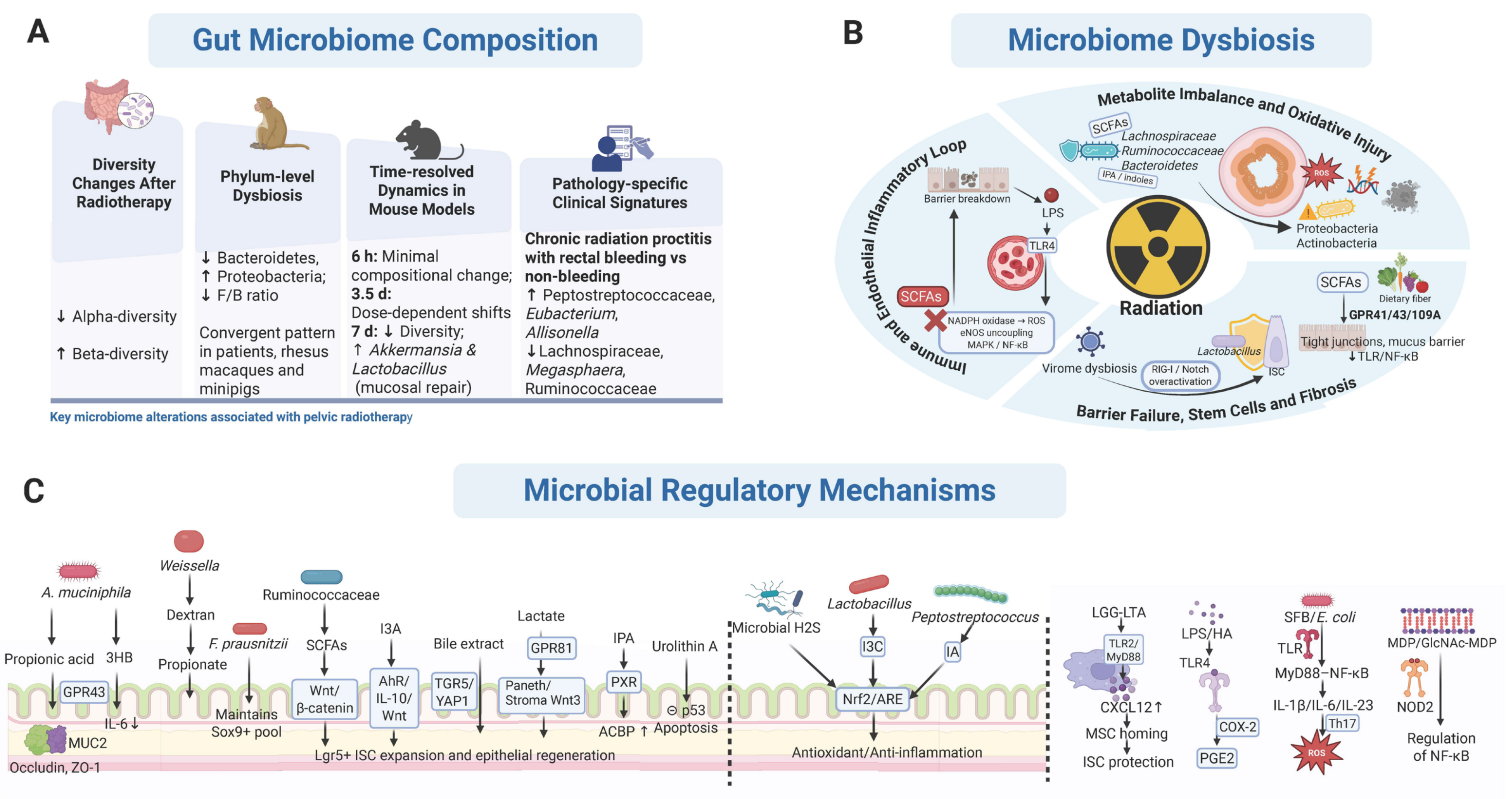
** Gut microbiome as a central regulatory network in RE.** (A) Changes in the gut microbiome after irradiation. (B) Radiation-induced dysbiosis sustains barrier failure, inflammation and impaired tissue repair. (C) Commensal microbes modulate post-irradiation gut barrier, survival and inflammatory circuits. 3-HB, 3-hydroxybutyrate; ACBP, acyl-CoA-binding protein; AhR, aryl hydrocarbon receptor; ARE, antioxidant response element; F/B ratio, Firmicutes/Bacteroidetes ratio; COX-2, cyclooxygenase-2; CXCL12, C-X-C motif chemokine ligand 12; *E. coli*, *Escherichia coli*; GPR43, G protein-coupled receptor 43; GPR81, G protein-coupled receptor 81; GPBAR1/TGR5, G protein-coupled bile acid receptor 1; H₂S, hydrogen sulfide; I3A, indole-3-aldehyde; I3C, indole-3-carbinol; IA, indoleacrylic acid; IL-1β, interleukin-1 beta; IL-6, interleukin-6; IL-10, interleukin-10; IPA, indole-3-propionic acid; ISC, intestinal stem cell; LGG, *Lactobacillus rhamnosus* GG; LGR5, leucine-rich repeat-containing G protein-coupled receptor 5; LPS, lipopolysaccharide; LTA, lipoteichoic acid; MAPK, mitogen-activated protein kinase; MDP, muramyl dipeptide; MSC, mesenchymal stem cell; MUC2, mucin 2; MyD88, myeloid differentiation primary response 88; NF-κB, nuclear factor kappa-B; NOD2, nucleotide-binding oligomerization domain-containing protein 2; NOX2, NADPH oxidase 2; Nrf2, nuclear factor erythroid 2-related factor 2; PGE2, prostaglandin E2; ROS, reactive oxygen species; SCFAs, short-chain fatty acids; SFB, segmented filamentous bacteria; TLR, Toll-like receptor; TLR2, Toll-like receptor 2; TLR4, Toll-like receptor 4; YAP1, Yes-associated protein 1; ZO-1, zonula occludens-1.

As the largest microbial ecosystem in the human body, the gut microbiome has been increasingly associated with, and may contribute to, the onset, progression and outcomes of RE. Multiple clinical studies using high-throughput 16S rRNA gene sequencing have shown that radiotherapy significantly alters microbial diversity and community structure. Diversity exhibits a dual pattern. Patients with RE show a marked reduction in alpha diversity (Simpson and Shannon indices), alongside a significant increase in beta diversity, indicating both loss of taxa and restructuring of the overall community^14^. Phylum-level dysbiosis shows cross-species consistency. The most characteristic change is a consistent decrease in Bacteroidetes coupled with an expansion of Proteobacteria. In patients receiving radiotherapy, 16S rRNA gene profiling indicates a decline in the Firmicutes/Bacteroidetes (F/B) ratio after treatment, correlating with dysbiosis and tissue injury; however, the F/B ratio should be interpreted only as a broad ecological descriptor rather than a standalone predictive biomarker^15^. Studies in two large animal models (rhesus macaques and minipigs) further confirm that, despite baseline inter-species differences, radiation induces highly convergent phylum-level shifts: decreased Bacteroidetes and increased Proteobacteria, whereas changes in Firmicutes vary by irradiation regimen and host species^16^. Time-resolved and pathology-specific shifts furnish candidate clinical biomarkers. In mouse irradiation models, microbiome dynamics are distinctly phase-dependent: during the prodromal phase (6 hours post-irradiation), changes are minimal; in the critical phase (3.5 days), composition closely tracks radiation dose; by the recovery phase (7 days), overall diversity declines, but the relative abundance of genera such as *Akkermansia* and *Lactobacillus* increases, potentially contributing to mucosal repair^17^. Clinically, patients with chronic radiation proctitis and rectal bleeding show distinct microbiome signatures compared with non-bleeding counterparts: enrichment of Peptostreptococcaceae, *Eubacterium* and *Allisonella*, with reduced Lachnospiraceae, *Megasphaera* and Ruminococcaceae—features that may serve as microbial markers for bleeding risk stratification and disease monitoring^18^ (Figure 2A).

However, direct comparisons across studies should be made with caution. Existing cohorts differ substantially in patient population, tumor type, irradiation site and treatment strategy, including cervical, prostate, rectal, endometrial and anal cancers treated with radiotherapy alone, concurrent chemoradiotherapy, postoperative adjuvant radiotherapy or radionuclide therapy. Radiation field, intestinal dose exposure, concurrent chemotherapy, antibiotic use, diet and comorbidities can all markedly reshape the gut microbiome. These variables may partly explain why the direction of change in specific taxa is not always consistent across studies. For example, *Faecalibacterium*, *Roseburia* and some *Clostridia* are generally regarded as SCFA producers with barrier-protective functions, yet in some clinical cohorts these taxa or related enterotypes have been associated with more severe radiation-induced gastrointestinal toxicity. Similarly, reported changes in the F/B ratio vary across studies, and this ratio alone has limited predictive value for RE. These inconsistencies suggest that the effects of putatively protective or deleterious taxa are context dependent and should not be extrapolated without considering tumor type, radiation dose, sampling time point, sequencing approach, diet and medication exposure.

Collectively, these human and animal data indicate that radiation induces characteristic, phase-dependent patterns of gut dysbiosis that actively shape the onset, progression and clinical manifestations of RE, while simultaneously offering candidate microbial biomarkers for risk stratification and longitudinal disease monitoring.

### 2.2 Microbiome dysbiosis tightly links to pathophysiological mechanisms

Under radiation-induced oxidative injury and cell-death programs, both animal and clinical studies consistently report reduced microbial diversity, depletion of beneficial and SCFA-producing members, a decrease in the F/B ratio, and expansion of pathobionts (for example, Proteobacteria and Actinobacteria). These shifts may increase endotoxin burden and activate inflammatory pathways, thereby potentially establishing a self-reinforcing “radiation-oxidative injury-barrier disruption-dysbiosis” positive feedback loop^19^. Notably, *Akkermansia* exhibits a biphasic, niche- and dose-dependent behavior: several studies show its depletion, and that supplementation or upregulation of its metabolite propionate can strengthen tight junctions and the mucus layer via GPR43, thereby attenuating radiation-induced intestinal injury; conversely, in acute high-dose models, *Akkermansia* has been reported to bloom post-irradiation, deplete mucin, and promote pathogenic adhesion and inflammation, thereby exacerbating injury^20^. In parallel, survivor-type models suggest that taxa enriched after irradiation (for example, Lachnospiraceae and Enterococcaceae) and their metabolites (propionate; indole pathway products such as I3A and kynurenic acid) mitigate DNA damage and ROS and promote hematopoiesis and mucosal repair. The tryptophan metabolite indole-3-propionic acid (IPA) confers systemic radioprotection via the pregnane X receptor (PXR)/acyl-CoA-binding protein (ACBP) axis; these interactions unite microbes, metabolites, redox homeostasis, and cell-death programs into an integrated network^21^.

In the setting of endothelial injury and microcirculatory dysfunction, barrier breakdown and translocation of bacterial products into the bloodstream may activate Toll-like receptor 4 (TLR4)-dependent NADPH oxidase-ROS production, eNOS uncoupling, and MAPK/NF-κB signaling, which together perpetuate endothelial dysfunction, microcirculatory derangement, dysbiosis, and endotoxemia/inflammation, although direct validation of this pathway in RE remains limited^22^. Microbial metabolites are both targets and modulators within this loop: SCFAs can restore eNOS and mitochondrial function in endothelial cells, lower ROS and dampen inflammation, thereby offering a means to interrupt the cycle; additionally, the commensal-derived SCFA valerate (pentanoate) confers systemic and gastrointestinal radioprotection *in vivo* and reshapes community composition^23^.

With respect to impaired repair, stem-cell dysregulation and chronic fibrosis, SCFAs act via GPR41, GPR43 and GPR109A and through histone deacetylase (HDAC) inhibition to reinforce tight junctions and the mucus barrier, and to restrain TLR/NF-κB amplification, thereby alleviating mucosal inflammation and promoting barrier reconstruction. Dietary fiber elevates SCFAs and other co-metabolites, which can attenuate gastrointestinal toxicity from radiotherapy while potentially enhancing anti-tumor efficacy^24^. The microbiota also directly influences epithelial stem-cell lineages: *Lactobacillus* promote ISC proliferation and barrier repair via the IL-22/STAT3 axis. Beyond bacteria, phage (virome) dysbiosis can suppress regeneration by overactivating RIG-I/Notch signaling within ISCs. Fecal virome transplantation from healthy donors partially restores stem-cell proliferation and differentiation, implicating multiple microbial compartments in the regulation of post-irradiation repair^25^. Collectively, the failed repair-stem cell dysregulation-chronic fibrosis axis is coupled to microbiome/virome disruption, metabolite depletion, and inflammatory amplification, defining a targetable network amenable to dietary fiber, probiotics/synbiotics, microbiota/virome transplantation, and metabolite supplementation^26^. It is worth noting that the roles of the gut mycobiome and broader virome in RE remain poorly defined, with current evidence remaining limited, preliminary and largely indirect, thereby highlighting the need for future studies integrating bacterial, fungal and viral microbiome datasets to better delineate host-microbiome interaction networks in RE.

Accordingly, dysbiosis should not be regarded solely as a downstream consequence of radiation injury; rather, it may contribute to the amplification and persistence of pathology: (1) Metabolite imbalance weakens endogenous protection. Post-irradiation loss of protective metabolites produced by beneficial taxa diminishes intrinsic anti-inflammatory, antioxidant and reparative capacity. (2) Immune dysregulation fuels an inflammatory vicious cycle. Overgrowth of pathobionts (for example, Proteobacteria) and their cell-wall components chronically activate TLR-MyD88-NF-κB signaling, escalating inflammation and reinforcing a loop of “dysbiosis-heightened inflammation-tissue injury-further dysbiosis”^27^,^28^. (3) Barrier disruption promotes bacterial translocation. Depletion of beneficial genera (for example, *Lactobacillus*, *Bifidobacterium*) thins the protective mucus layer; dysbiosis-driven signals downregulate tight-junction proteins (ZO-1, occludin), increasing permeability and facilitating translocation of bacteria and toxins, which triggers local and systemic inflammation^29^. Radiation also perturbs the physical barrier to skew microbe-immune crosstalk: loss of REGγ reduces goblet cells and thins the mucus layer post-irradiation, enabling closer bacterial-epithelial contact and exacerbating inflammation. It is a phenotype closely tied to compositional shifts, particularly loss of probiotics such as *Lactobacillus* and *Bifidobacterium*^30^. Moreover, radiation-induced NF-κB activation upregulates miR-221/222 and suppresses Syndecan-1, leading to ZO-1 and occludin downregulation and barrier failure. Dysbiosis magnifies these changes, reflected by elevated circulating soluble Syndecan-1 and inflammatory cytokines (TNF-α, IL-1β) in patients with RE^29^. It should be emphasized that most clinical microbiome studies in RE are based on associations between fecal sequencing profiles and symptom scores or toxicity grades. Antibiotic depletion, fecal microbiota transplantation, specific strain supplementation and metabolite rescue experiments in animals provide stronger evidence for causality. Accordingly, clinical causality remains to be established through prospective, mechanism-guided interventional studies (Figure 2B).

### 2.3 Insights into microbiome-based regulatory mechanisms: emerging perspectives

Microbial metabolites play pivotal roles in the progression of RE. These small molecules act across multiple signaling axes to coordinate barrier function, inflammatory responses and stem-cell regeneration, forming a multifaceted interactive network. The SCFA-GPR axis is a meaningful regulator of barrier integrity. SCFAs, the principal products of microbial fermentation of dietary fiber, are central to gut homeostasis. Radiotherapy significantly reduces SCFA production; notably, depletion of mucosa-associated, SCFA-producing genera correlates with radiation injury of the anterior rectal mucosa^31^. Propionate and butyrate exert particularly salient protective effects. *Akkermansia muciniphila* protects the gut against irradiation by secreting propionate, which engages G-protein-coupled receptor 43 (GPR43) on intestinal epithelial cells, enhances histone acetylation and upregulates tight-junction proteins (occludin, ZO-1) and mucin (MUC2), thereby strengthening epithelial barrier integrity^20^. In patients, the abundance of *A. muciniphila* inversely correlates with the duration of diarrhea; supplementation improves survival and preserves intestinal architecture in mice^32^. Recent work also indicates that *Faecalibaculum rodentium* is a radioprotective keystone species whose depletion coincides with reduced butyrate and increased ionizing radiation sensitivity. Mechanistically, butyrate curtails ERK-driven nuclear translocation of PKM2, thereby attenuating p53/BAX-dependent apoptosis in hematopoietic stem and progenitor cells (HSPCs)^33^. 3-hydroxybutyrate (3HB) represents a second protective route. 3HB ameliorates radiation proctopathy by blocking GPR43-mediated IL-6 signaling. As an important source of 3HB, *A. muciniphila* declines after irradiation, with 3HB levels inversely associated with IL-6 expression; supplementation with 3HB or *A. muciniphila* lowers GPR43 and IL-6 expression and improves clinical symptoms and histopathology^34^.

Additional microbiota-derived factors fortify the barrier. Dextran produced by *Weissella* increases propionate and promotes mucosal barrier function^35^. Prophylactic administration of *Faecalibacterium prausnitzii* protects the colonic barrier by maintaining the Sox-9+ stem/progenitor pool and stimulating crypt epithelial proliferation^36^. *Lactobacillus rhamnosus* GG (LGG) safeguards the gut via dual mechanisms: suppression of JAK2/STAT3 signaling to reduce pro-inflammatory cytokines, and activation of a TLR2/COX-2-dependent pathway that drives COX-2+ mesenchymal stem cells to home towards the ISCs niche, creating a protective microenvironment^37^,^38^.

The Wnt signaling network governs ISC dynamics. The Wnt/β-catenin pathway is essential for ISC self-renewal and differentiation and is finely tuned by microbial metabolites. SCFAs produced by the Ruminococcaceae family activate Wnt/β-catenin signaling, enhance *Lgr5*^+^ ISC expansion and promote epithelial regeneration^39^,^40^. The microbial indole-3-aldehyde (I3A) activates the aryl hydrocarbon receptor (AhR)/IL-10/Wnt axis to stimulate epithelial proliferation, mitigate mucosal injury and maintain barrier integrity^41^. As an environmental sensor, AhR constrains Wnt-β-catenin signal strength by modulating RNF43 and ZNRF3, thereby preserving ISC homeostasis and barrier function^42^. By contrast, under pathological conditions FOXQ1 upregulates SIRT1 and promotes β-catenin nuclear translocation, heightening stemness and radioresistance in colorectal cancer cells while reshaping the microbiota, illustrating the pathogenicity of Wnt pathway dysregulation^43^. The bile acid-TGR5 axis underpins metabolism and regeneration. Untargeted metabolomics reveals significant post-irradiation reductions in secondary bile acids (for example, deoxycholic acid and lithocholic acid), which possess anti-inflammatory properties; their loss may worsen radiation-induced inflammation^44^. Supplementation with bile extract or lithocholic acid promotes ISC proliferation and intestinal regeneration by modulating G-protein-coupled bile acid receptor 1 (TGR5) and Yes-associated protein 1 (YAP1)^45^. Lactate provides an additional regenerative cue. By engaging GPR81, lactate induces Paneth and stromal cells to produce Wnt3, accelerating ISC-mediated epithelial development; pre-feeding lactate protects mice from chemotherapy- and radiation-induced intestinal injury^46^.

Beyond these major axes, several specialized metabolites confer added protection. Indole-3-propionic acid (IPA) activates PXR to sustain ACBP expression, thereby protecting the gut from radiation injury^47^. Urolithin A (UroA) has shown radioprotective activity by inhibiting p53-mediated apoptosis and remodeling the gut microbiota; in an immunosuppressive tumor microenvironment, UroA reduces tumor-associated macrophages and enhances the efficacy of radiotherapy^48^. The Nrf2 antioxidant pathway is involved in countering radiation-induced oxidative injury. Microbiota-derived hydrogen sulphide (H_2_S) directly activates the Keap1/Nrf2/ARE cytoprotective axis, upregulating antioxidant proteins and glutathione to mitigate damage in normal tissues. Acid-producing taxa such as *Lactobacillus acidophilus* catalyze the conversion of dietary indole-3-carbinol (I3C) into bioactive anticancer compounds that lessen radiation injury via Nrf2-dependent mechanisms. Indoleacrylic acid (IA) produced by commensal *Peptostreptococcus* likewise suppresses inflammation and strengthens the barrier through Nrf2-ARE activation^49^. KEGG analyses further implicate multiple microbiome-related metabolic routes in RE, including arginine, arachidonic acid and linoleic acid metabolism^50^.

The gut microbiota also engages host immunity through multiple pattern-recognition receptors (PRRs). Under physiological conditions, beneficial microbes elicit TLR-mediated protective responses that confer radioprotection. LGG releases lipoteichoic acid (LTA), a TLR2 agonist that binds TLR2 on subepithelial macrophages, triggering MyD88-dependent signaling and robust CXCL12 secretion. The resulting chemokine gradient actively recruits mesenchymal stem cells to the crypt niche, thereby shielding ISCs from radiation injury^38^,^51^. Lipopolysaccharide (LPS) and hyaluronan provide radioprotection via TLR4-COX-2 activation, boosting prostaglandin E2 (PGE2) production and protecting crypt cells from radiation-induced apoptosis^27^. When irradiation drives dysbiosis, TLR signaling shifts towards inflammation. Radiation-induced overgrowth of segmented filamentous bacteria (SFB) and *Escherichia coli* activates TLR-MyD88-NF-κB signaling, enhancing production of IL-1β, IL-6 and IL-23. This promotes Th17 polarization and accumulation; Th17-derived IL-17 and granulocyte-macrophage colony-stimulating factor (GM-CSF) in turn expand and activate neutrophils, foster oxidative stress and exacerbate dysbiosis, closing a vicious cycle^28^. The microbiota can also modulate RE via NOD2: muramyl dipeptide (MDP) and N-acetylglucosamine-MDP, generated by *Enterococcus* peptidoglycan hydrolases, engage NOD2 to activate NF-κB and regulate inflammation^52^.

Taken together, these findings suggest that microbiome-related signaling in RE is better understood as a hierarchical, phenotype-linked network rather than a set of equivalent pathways. Based primarily on preclinical evidence, the SCFA-GPR/HDAC axis represents the most direct microbiome-dependent barrier-protective mechanism, maintaining tight-junction integrity, limiting mucosal inflammation and supporting intestinal and hematopoietic stem/progenitor cell survival. A second major module is the microbial metabolite-regulated regenerative axis, which includes Wnt/β-catenin, AhR/IL-10/Wnt, bile acid-TGR5/YAP1 and lactate-GPR81-Wnt3 signaling and collectively governs ISC maintenance, epithelial regeneration and post-irradiation repair. In contrast, TLR-MyD88-NF-κB signaling is best positioned as a downstream inflammatory amplifier that becomes prominent when irradiation-induced barrier failure and dysbiosis increase exposure to microbial products, thereby connecting acute mucosal inflammation with persistent immune activation. Other pathways, including Nrf2, PXR/ACBP, cGAS-STING and metabolite-specific mechanisms, should be viewed as context-dependent modifiers or therapeutic entry points that intersect with these core processes. This hierarchy also helps align mechanisms with clinical phenotypes: SCFAs depletion and barrier failure are most closely associated with acute diarrhea and mucosal injury; impaired regenerative signaling may contribute to delayed repair and acute-to-chronic transition; and sustained inflammatory amplification together with pro-fibrotic remodeling is more relevant to chronic bleeding, fibrosis and stricture formation (Table 1 and Figure 2C).

Nevertheless, most mechanistic studies are based on mouse or rat models of acute, high-dose irradiation, including total abdominal irradiation, total-body irradiation, and localized intestinal irradiation. Although these models are valuable for elucidating epithelial injury, inflammatory activation, and microbe-metabolite interactions, they differ substantially from clinical settings that involve fractionated radiotherapy, intensity-modulated radiotherapy, or concurrent chemoradiotherapy. Therefore, findings from animal studies should be interpreted primarily as mechanistic insights, and validation in human cohorts remains essential.

## 3. Gut microbiota characteristics in RE across different tumor types

**Figure 3:**
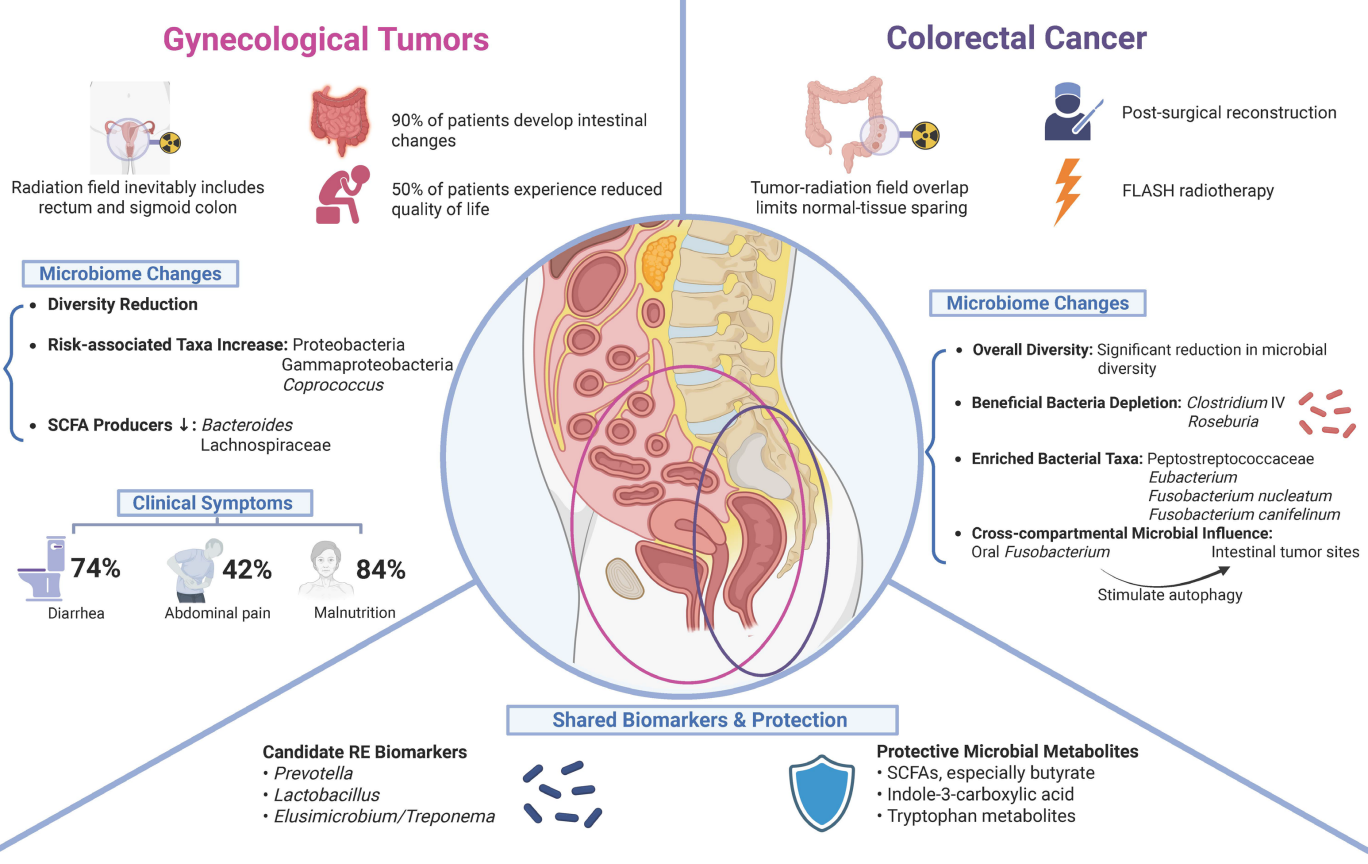
** Tumor-specific landscapes of RE.** In colorectal cancer, radiation-associated dysbiosis features loss of SCFA producers and enrichment of anaerobic, pro-fibrotic and *Fusobacterium*-dominated communities linked to symptoms, fibrosis and reduced radiosensitivity. In gynecological tumors, the gut microbiome exhibits reduced diversity with Proteobacteria and Gammaproteobacteria increases and *Bacteroides* decreases; baseline *Coprococcus* enrichment identifies patients at higher enteritis risk.

The gut microbiome in RE is not a homogeneous entity. Microbial composition and inferred function in RE vary substantially with primary tumor type, anatomical location and radiation regimen, and these tumor- and treatment-specific microbiome configurations are closely linked to differences in the clinical presentation and severity of RE, providing a basis for personalized risk stratification and precise prevention strategies (Figure 3).

### 3.1 Colorectal tumors

Colorectal tumor-associated RE has unique anatomical and treatment characteristics. In standard neoadjuvant regimens for rectal cancer, pelvic radiation fields routinely encompass the mesorectum, presacral and internal iliac nodes, and, in T4 or distal tumors, extend anteriorly to cover the external iliac or even inguinal basins, which tends to bring additional small bowel and the anal canal into the high-dose region^53^. Because the tumor often overlaps the irradiation field, sparing normal tissue is technically challenging. Consequently, pelvic radiotherapy in colorectal tumors may promote epithelial injury, barrier disruption, inflammatory activation, and local microbial remodeling.

Existing evidence indicates that patients with colorectal cancer (CRC) already exhibit marked intestinal dysbiosis at the time of diagnosis, characterized by reduced overall microbial diversity and richness, enrichment of pro-inflammatory and opportunistic pathogenic taxa, and depletion of butyrate-producing “beneficial” bacteria. Neoadjuvant radiochemotherapy (NRCT) does not substantially alter the global diversity or overall structure of the gut microbiota, but can decrease the relative abundance of potentially pathogenic genera such as *Fusobacterium*, *Escherichia* and *Klebsiella*, while increasing *Bifidobacterium*, a shift that is considered relatively favorable^54^. Analyses of clinical specimens suggest that the intratumoral microbiome may have predictive value for radiotherapeutic response. Specifically, the presence of *Fusobacterium canifelinum* has been associated with unfavorable prognosis, whereas microbiota-directed interventions, such as antibiotics or probiotics, may enhance radiosensitivity by modulating tumor-associated microbial communities^55^. In murine models, oral inoculation with *Fusobacterium nucleatum* followed by irradiation allows this species to translocate from the oral cavity to CRC lesions and attenuate the efficacy of radiotherapy, whereas metronidazole effectively suppresses *F. nucleatum* colonization and restores radiosensitivity. These findings highlight the critical role of the oral-gut microbiota axis in shaping responses to radiation therapy and suggest that optimizing oral hygiene and oral microbiome status may represent an important component of comprehensive management in CRC patients undergoing radiotherapy^56^.

### 3.2 Gynecological tumors

Radiotherapy for gynecological malignancies has distinct anatomical and physical characteristics: target positioning is markedly influenced by bladder and rectal filling, and pelvic organs such as the uterus and cervix can undergo substantial displacement and deformation, in contrast to the relatively fixed targets seen in most thoracic and head-and-neck tumors. At the same time, gynecological pelvic irradiation typically involves large fields and high doses, and, combined with generally long patient survival, this results in a high incidence of multisystem chronic toxicities^57^. Moreover, fields for cervical and endometrial cancer inevitably include the rectum and sigmoid colon^58^.

Even with intensity-modulated radiotherapy (IMRT), about 90% of patients experience persistent intestinal changes and 50% report marked deterioration in quality of life^14^. During cervical cancer radiotherapy, the incidence of diarrhea and abdominal pain rises weekly, peaking at treatment end (diarrhea 74%, abdominal pain 42%)^59^. Nutritional risk escalates throughout treatment: by completion, up to 84% exhibit deficiency, with significant declines in body weight, skeletal muscle mass and serum albumin^60^. In older women treated for endometrial cancer, risks of constipation (HR=2.27), abdominal pain (HR=2.94) and fecal incontinence (HR=1.96) exceed those in cancer-free peers and persist for at least 5 years^61^. High-throughput sequencing shows marked loss of gut microbial diversity during therapy, with increased Proteobacteria and Gammaproteobacteria and decreased *Bacteroides*. Pre-treatment microbiota can predict enteritis risk; for example, higher baseline abundance of *Coprococcus* associates with severe RE^14^. Diversity tightly relates to chemoradiation toxicity: the Shannon index fell from baseline (2.9±0.5) to week 5 (2.49±0.7), and patients with higher diversity generally experienced fewer gastrointestinal symptoms^62^.

Although reduced diversity and depletion of SCFA-producing bacteria are shared features, the gut microbiota associated with RE displays tumor-type-specific compositional and functional profiles shaped by the underlying malignancy and radiation strategy, indicating that we should take tumor-type specificity into account rather than focusing solely on inflammation itself. These cancer-specific microbiome signatures highlight the need for tumor-type-specific microbial risk assessment and tailored intervention strategies.

## 4. Gut microbiota characteristics in acute and chronic RE

**Figure 4:**
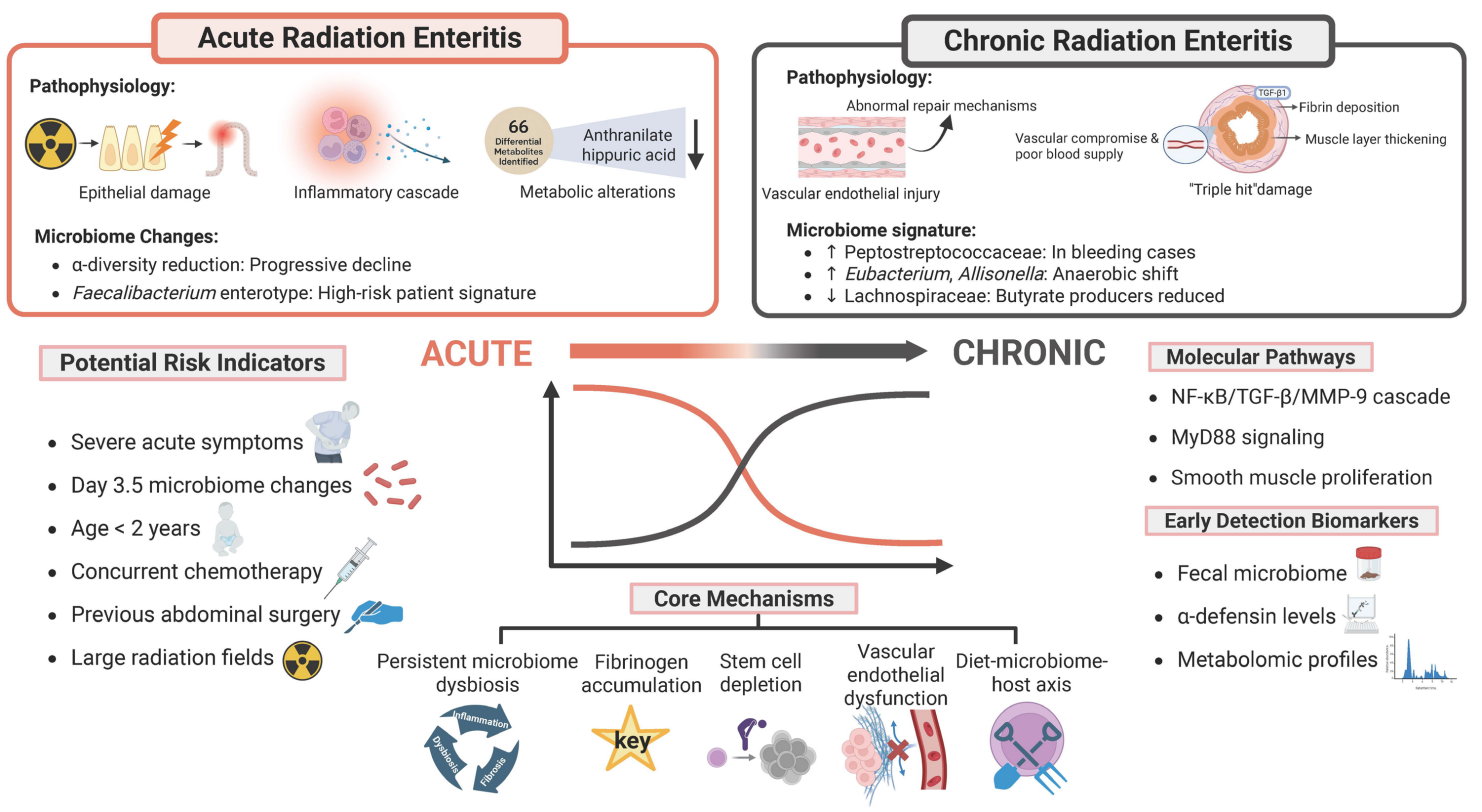
** Features of acute and chronic RE and mechanisms of transition.** The figure illustrates microbiome-driven transitions from acute inflammatory dysbiosis to chronic, anaerobic, pro-fibrotic community states. Core mechanisms link acute injury to chronic disease through sustained dysbiosis, impaired regeneration, fibrosis, vascular dysfunction and disrupted diet-microbiome-host metabolism. Representative interventions are shown on both sides. α-diversity, alpha diversity; MMP-9, matrix metalloproteinase-9; MyD88, myeloid differentiation primary response 88; NF-κB, nuclear factor kappa B; TGF-β, transforming growth factor beta.

The gut microbiota characteristics in RE not only vary depending on the tumor type and corresponding radiation regimen but are also closely associated with distinct stages of RE progression. RE can be clearly divided into acute and chronic phases based on timing and pathophysiology. According to expert consensuses and clinical guidelines from various countries, including the United States, Europe, and Australia, as well as the 2021 Chinese expert consensus, a 3-month interval after radiotherapy is commonly used in clinical practice to distinguish acute from chronic RE. This cutoff has also been widely adopted in clinical and translational studies. The two differ fundamentally in clinical phenotype, histological underpinnings and putative microbiome signatures. Crucially, deciphering the roles of multiple factors, including the gut microbiome, in the transition from acute to chronic disease is key to preventing long-term severe complications (Figure 4).

### 4.1 Features of acute phase

Acute RE typically emerges during radiotherapy or within weeks after completion. Pathologically, ionizing radiation inflicts direct, rapid injury on the mucosa—most prominently on crypt stem cells with high mitotic rates. This damage drives extensive apoptosis of clonogenic crypt cells, disrupts the epithelial renewal cycle and triggers downstream cascades. Histology shows villous atrophy, blunting and fusion; crypt disarray or loss; marked depletion of goblet cells; submucosal oedema; and diffuse inflammatory infiltrates dominated by neutrophils. The resultant physical barrier breakdown increases intestinal permeability and elicits a strong innate immune response with surges of pro-inflammatory cytokines such as IL-1β, IL-6 and TNF-α^63^-^65^. Clinically, patients develop diarrhea, abdominal pain, nausea, vomiting and tenesmus, with severity closely linked to cumulative dose and often peaking at treatment end. In a cervical-cancer cohort, diarrhea rose from 28% in week 2 of radiotherapy to 74% by treatment completion^59^. Time-series analyses identify week 4 post-radiotherapy as a critical inflection point: levels of arachidonic acid and other metabolites reach a nadir and then rebound, consistent with the onset of gut self-regulatory responses^59^. In parallel, nutritional status deteriorates; by treatment end, up to 84% of patients exhibit malnutrition with significant declines in body weight, skeletal muscle mass and serum albumin^60^.

The microbiome shifts sharply in the acute phase, with reduced diversity and dysbiosis. Changes scale with radiation dose: at a critical window around day 3.5 post-exposure, the relative abundance of Proteobacteria and the genus cluster *Escherichia-Shigella* increases dose-dependently, while beneficial *Lactobacillus murinus* decreases^17^. We speculate that such dysbiosis likely arises on the background of radiation-induced epithelial and mucus barrier disruption, which facilitates overgrowth and mucosal adhesion of facultative pathobionts and enhances exposure of pattern-recognition receptors to luminal pathogen-associated molecular patterns, thereby amplifying mucosal inflammation and further impairing barrier integrity. As many of these depleted taxa are SCFA producers that support epithelial energy supply and tight-junction maintenance, their early loss is expected to weaken barrier repair and promote a pro-inflammatory milieu. Serum metabolomics in an acute rat model identified 66 significantly altered metabolites, with anthranilic acid and hippuric acid showing the most pronounced decreases; the lipid metabolite linoleic acid emerged as a candidate biomarker of high nutritional risk^44^,^60^. Clinically, fecal microbiome profiling shows promise as a biodosimeter for radiation injury. Thus, acute RE should be viewed not merely as a process of microbial compositional change, but as a reciprocal loop in which radiation-induced barrier disruption drives dysbiosis, while dysbiosis further aggravates mucosal inflammation and barrier failure. Although direct mechanistic evidence for microbiome-mediated pathways in acute RE is still limited, based on the above findings we hypothesize that acute-phase barrier disruption and dysbiosis are mechanistically linked and together shape the inflammatory and metabolic landscape that determines progression versus resolution of injury in later phases.

### 4.2 Features of chronic phase

In contrast, chronic RE is a delayed injury that can present months to up to three decades after therapy. Its pathological core is aberrant repair of early damage, culminating in progressive ischemic vasculopathy and tissue fibrosis^31^,^66^. Among patients receiving pelvic radiotherapy, incidence is approximately 5-20%^18^. TGF-β1, a key pro-fibrotic mediator, is activated in cascade to drive fibroblast proliferation and extracellular-matrix deposition^67^, accompanied by intimal hyperplasia and microvascular remodeling. This establishes a characteristic “triple hit” of the bowel wall, comprising excess fibrin deposition, pathological thickening of the muscularis and compromised vascular supply^66^, which together define the histological basis of chronic injury. These substrates produce a broad clinical spectrum dominated by altered bowel habits, persistent abdominal pain and intermittent bleeding. Unlike the inflammation-driven symptoms of the acute phase, chronic manifestations reflect permanent architectural change; roughly 50% of long-term survivors develop some form of chronic gastrointestinal dysfunction^31^. With advancing fibrosis, about 10% progress to strictures, fistulas or perforation, often requiring surgery^66^. Symptom timing shows a bimodal pattern: in some, symptoms track continuously with treatment, whereas in others they arise after a 6- to 24-month latency, underscoring the complex dynamics of fibrogenesis^67^.

Microbiome studies reveal deep ecological shifts in chronic disease that likely participate in maintenance and progression rather than being mere sequelae of radiation. This distinction may influence reparative capacity: long-term depletion of classical butyrate-producing taxa, including *Clostridium* cluster IV, *Roseburia* and *Phascolarctobacterium*, correlates positively with progression of intestinal fibrosis and loss of butyrate (which serves as both a primary energy source for colonocytes and an anti-inflammatory and epigenetic modulator) may contribute to the inflammation-fibrosis cycle by permitting sustained TGF-β/SMAD-dependent myofibroblast activation and collagen deposition^68^. Deeper mucosal profiling shows significant reductions in homeostatic cytokines that sustain barrier integrity, including IL-7, IL-12/IL-23p40, IL-15 and IL-16^31^, which we speculate reflects disruption of microbial ecological niches following acute barrier injury, leading to persistent immune dysregulation that in turn fuels chronic inflammation and progressive fibrosis.

### 4.3 Hallmarks of the acute-to-chronic transition

The transition from acute to chronic RE arises from interlocking pathophysiological processes. In the acute phase, abrupt epithelial barrier loss, mucosal cytokine shifts and dose-dependent dysbiosis create a highly inflamed and metabolically perturbed luminal environment; early expansion of facultative pathobionts and depletion of SCFA producers such as classical butyrate-producing taxa may weaken barrier repair and prolong immune activation. Persistent dysbiosis may be an important contributor to a sustained low-grade inflammatory milieu that is increasingly recognized as a legacy of these early ecological disturbances rather than a de novo phenomenon. At the molecular level, fibrinogen accumulation in intestinal tissues is pivotal; its deficiency markedly attenuates both early and delayed injury and reduces abnormal smooth-muscle proliferation and TGF-β expression^69^. Beyond TGF-β signaling, Wnt/β-catenin dysregulation impairs stem-cell renewal, and persistent NF-κB activation maintains a chronically inflamed microenvironment. Vascular injury is likewise key: radiation-induced endothelial damage leads to microvascular occlusion and impaired angiogenesis, producing ischemic tissue injury that amplifies fibrosis. Critically, repetitive acute insults deplete the intestinal stem-cell pool, limiting long-term epithelial regeneration^26^.

Integrating microbial and host factors, we hypothesize that early barrier failure and microbiome perturbation may increase the risk of chronic disease. These changes may promote a feed-forward loop involving dysbiosis, cytokine dysregulation and pro-fibrotic signaling, including TGF-β/SMAD and NF-κB pathways. Over time, this process may contribute to fibrosis and vascular remodeling. For risk stratification, the severity of acute symptoms strongly predicts chronic progression; all patients who later developed delayed RE had previously exhibited pronounced acute reactions^70^. Microbiome signatures can also serve as predictors, especially changes at the critical ~3.5-day timepoint post-irradiation rather than at 6 hours (prodrome)^17^. Additional high-risk groups include patients with significant comorbidities and those receiving high doses and/or large irradiation fields. In children, younger age (particularly under 2 years), prior abdominal surgery and concurrent chemoradiation, especially with anthracyclines (for example, doxorubicin), increase risk^70^.

Technical differences further complicate interpretation of phase-specific microbiome signatures. Existing studies differ in sequencing platform, 16S rRNA variable region, bioinformatic workflow and taxonomic database. Most rely on 16S rRNA sequencing, which often provides genus-level or coarser taxonomic resolution and has limited capacity to resolve strain-level variation or functional genes. Functional inference using tools such as PICRUSt can generate useful hypotheses but cannot replace shotgun metagenomics, metatranscriptomics, metabolomics or culture-based validation. These limitations should be considered when interpreting acute- and chronic-phase biomarkers or comparing microbial signatures across studies.

## 5. Systemic effects of radiotherapy: microbiome-mediated inter-organ crosstalk

**Figure 5:**
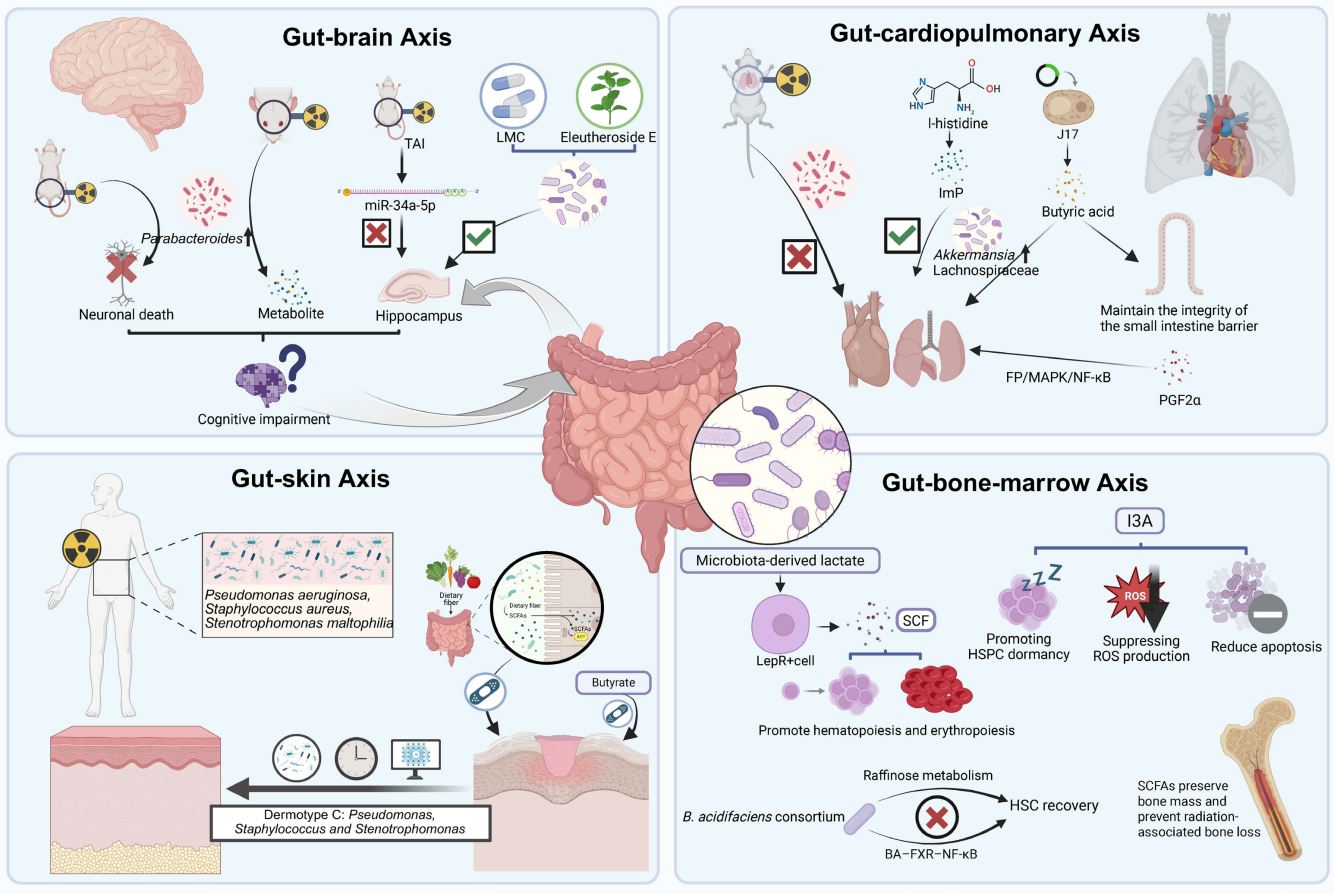
** Microbiome-mediated gut-organ axes underlying systemic effects of radiotherapy.** Radiotherapy elicits systemic effects via microbiome-mediated gut-organ axes, revealing actionable targets. Gut-brain axis: After irradiation, disruption of the gut-brain axis links microbial shifts with miR-34a-5p/BDNF alterations and candidate neuroprotective interventions. Gut-cardiopulmonary axis: A gut-cardiopulmonary axis emerges whereby microbial metabolites and engineered strains modulate barrier integrity, immune balance and cardiopulmonary injury. Gut-skin axis: In radiation-induced dermatitis, distinct microbiome “dermotypes” marked by opportunistic taxa correlate with chronicity and delayed healing. Gut-bone marrow axis: Microbial metabolites and host mediators regulate post-irradiation hematopoiesis by promoting HSPC dormancy, survival and supportive niche signaling. TAI, total abdominal irradiation; BDNF, brain-derived neurotrophic factor; EE, Eleutheroside E; LMC, Lactobacillus microcapsules; ImP, imidazole propionate; HSPC, hematopoietic stem and progenitor cell; HSC, hematopoietic stem cell; LepR⁺, leptin receptor-positive; SCF, stem cell factor; TNF, tumor necrosis factor; RID, radiation-induced dermatitis.

The impact of the gut microbiome in RE in response to radiotherapy on the host does not represent an isolated event; through the gut-organ axes, which constitute a multilayered communication network, it systemically influences distant organ function. Here we outline the roles of the gut-brain, gut-cardiopulmonary, gut-skin, and gut-bone-marrow axes in microbiota-mediated radiation-associated injury. Because evidence specifically linking gut-organ axes to RE remains limited, this section presents the gut-organ axis framework as a conceptual and mechanistic extension derived from broader studies of radiation-associated systemic injury (Figure 5).

### 5.1 Gut-brain axis

The gut-brain axis denotes bidirectional communication between the central nervous system and the gastrointestinal tract, integrating the central nervous system, enteric and autonomic nervous systems, neuroendocrine, neuroimmune pathways and the gut microbiota with its metabolites^71^. Cognitive impairment after radiotherapy reflects not only direct cranial exposure but also distant effects mediated by the gut-brain axis, although current evidence comes almost entirely from preclinical rodent studies and varies considerably in mechanistic depth. In a mouse model with causal validation, total abdominal irradiation (TAI) impairs cognition through the miR-34a-5p/BDNF axis. TAI significantly elevates miR-34a-5p in the small intestine and peripheral blood while lowering hippocampal BDNF, resulting in cognitive deficits. Tail-vein delivery of a miR-34a-5p antagonist restores hippocampal BDNF and cognition and reverses dysbiosis, notably rebalancing Bacteroidetes and Firmicutes. Fecal microbiota transplantation (FMT) further confirms that microbiota changes directly contribute to cognitive impairment^72^. In contrast, other reports remain largely observational and have not yet been causally verified. In a rat model, a single 6 Gy dose markedly altered the microbiota, particularly increasing the genera *Parabacteroides*, *Sutterella* and *Desulfovibrio*, and induced neuronal death, reduced neuronal maturity and astrocyte activation, ultimately affecting exploratory behavior in rats. Although this regimen had limited impact on memory, hippocampal plasticity markers such as Nmda2 decreased, revealing a pathway by which pelvic irradiation influences neuronal plasticity via the gut-brain axis^73^. Conversely, cranial irradiation can perturb distant gut ecology and systemic metabolism, underscoring the bidirectional nature of this communication. In a mouse model, after 20 Gy X-ray cranial exposure, gut community structure shifts; at 2 weeks, *Muribaculum* and *Parabacteroides* increase, whereas by 4 weeks *Akkermansia* and *Helicobacter* decline. Brain irradiation can affect the intestinal flora and metabolome far away from the irradiation site through the gut-brain axis, and there are common pathways for metabolic changes in different organs, especially arginine and proline metabolism^74^.

Radioprotective strategies targeting this axis are emerging, though evidence to date is limited to mouse models. Eleutheroside E (EE) pretreatment mitigated radiation-induced cognitive dysfunction by remodeling the microbiota (increasing *Lactobacillus*, decreasing *Helicobacter*), modulating neurotransmitters and activating PKA/CREB/BDNF signaling^75^. *Lactobacillus* microcapsules (LMC) likewise alleviated radiation-related brain injury by improving the intestinal milieu, enriching Lachnospiraceae, *Blautia* and *Lactobacillus*, lowering inflammatory cytokines, ameliorating hippocampal pathology, reducing anxiety and enhancing memory^76^.

From the perspective of RE risk, the most important implication of brain irradiation studies is not that cranial radiation directly causes RE, but that cranial exposure can remotely remodel gut microbial communities. Several altered taxa have known links to intestinal barrier homeostasis. In particular, the decline of *Akkermansia* after cranial irradiation overlaps with observations from radiation-induced intestinal injury and radiation proctopathy, in which *Akkermansia*-derived metabolites support tight-junction integrity, mucus production and anti-inflammatory signaling. By contrast, changes in *Parabacteroides*, *Muribaculum* and *Helicobacter* are more context-dependent and should be interpreted cautiously. These findings suggest that cranial irradiation may generate a gut microbial state with potential relevance to intestinal inflammatory susceptibility, although direct evidence for cranial-irradiation-induced enteritis is still lacking.

### 5.2 Gut-cardiopulmonary axis

The gut-cardiopulmonary axis centers on the microbiota and its metabolites and links the gut with the cardiovascular and respiratory systems via immune, metabolic, neuroendocrine and barrier pathways. Shared features include dysbiosis and increased permeability driving systemic inflammation and endothelial dysfunction; circulating microbial metabolites shaping myocardial/vascular and pulmonary immune milieus; and reciprocal remodeling of gut ecology by cardiorespiratory disease through low perfusion, hypoxia, medications and diet^77^,^78^. Thoracic irradiation has also been shown to remodel the gut microbiota itself. Chest irradiation alters gut microbial composition in mice, and gut microbiota-derived prostaglandin F2α (PGF2α) can penetrate the intestinal barrier, enter the circulation and activate the FP/MAPK/NF-κB signaling pathway to mitigate lung injury^79^. Thoracic irradiation is further associated with a decrease in Firmicutes and an increase in Bacteroidetes^80^, and, in related models, gut microbiota attenuate acute LPS-induced lung damage via the TLR4/NF-κB pathway^81^.

Specific microbiota-derived metabolites confer radioprotection to the heart and lungs. Oral L-histidine prevents radiation-induced structural injury and collagen accumulation in the heart and lungs while preserving microbial balance, notably increasing *Akkermansia* and *Lactobacillus*; ImP acts downstream to inhibit pyroptotic cell-death programs in lung cells^82^. From the perspective of intestinal inflammatory risk, these findings are relevant because thoracic irradiation, although primarily causing lung or heart injury, may also generate gut microbial perturbations that overlap with enteritis-associated dysbiosis. In particular, depletion of Firmicutes may indicate a reduced capacity for SCFA-production, whereas changes in Bacteroidetes may reflect broader ecological imbalance. Conversely, preservation or enrichment of *Akkermansia* and *Lactobacillus* during radioprotective interventions suggests that microbial configurations associated with cardiopulmonary protection may also favor intestinal barrier integrity.

Taken together, these studies highlight a gut-cardiopulmonary axis in the context of radiotherapy and support microbiome-targeted radioprotective strategies. However, the available evidence is currently derived mainly from preclinical animal experiments. Therefore, it should not be interpreted as proof that thoracic irradiation directly causes RE. Rather, these findings indicate that extra-abdominal irradiation can induce gut microbial changes that overlap with microbial patterns implicated in intestinal inflammation, thereby suggesting a potential, hypothesis-generating link between thoracic irradiation-induced dysbiosis and intestinal inflammatory susceptibility.

### 5.3 Gut-skin axis

The gut-skin axis is a bidirectional network anchored by the gut microbiota and its metabolites, connecting gut and skin through immune-inflammatory, metabolic-endocrine, neuroendocrine and barrier-integrity pathways. Eubiosis supports skin barrier function and immune tolerance, whereas dysbiosis and increased gut permeability can drive systemic inflammation and endothelial/keratinocyte dysfunction, contributing to atopic dermatitis, psoriasis and acne; conversely, the skin microenvironment and dermatological therapies may also remodel the gut ecosystem^83^. Radiation-induced dermatitis (RID) is closely linked to local skin microbial dysbiosis and may also be influenced by the broader gut-skin axis. Radiotherapy significantly reshapes the skin microbiome; RID is associated with reduced microbial diversity, and microbial diversity has been reported to correlate positively with wound healing^84^. Compositionally, RID lesions are often dominated by opportunistic or inflammation-associated taxa such as *Klebsiella*, *Staphylococcus* or *Pseudomonas*; the co-occurrence of *Pseudomonas aeruginosa*, *Staphylococcus aureus* and *Stenotrophomonas maltophilia* correlates with chronicity, particularly in diabetes^84^. A microbiome-based “skin-type” classifier identifies a type C pattern (dominated by *Pseudomonas*, *Staphylococcus* and Stenotrophomonas) that has been associated with delayed healing and may aid risk stratification^84^. Some of these taxa, particularly *Klebsiella*, have established roles as intestinal pathobionts in ‌Inflammatory Bowel Disease‌-like inflammation; however, the available evidence mainly concerns local skin microbiome alterations and does not demonstrate that skin irradiation induces gut dysbiosis, enteritis, or RE.

Gut-derived SCFAs are key mediators of epithelial barrier integrity, including in the skin. Dietary-fiber-derived SCFAs, especially butyrate, promote keratinocyte differentiation and barrier-protein expression. Orally administered butyrate rapidly reaches skin, enhances mitochondrial fatty-acid β-oxidation in keratinocytes and improves barrier function^85^. However, most mechanistic insights and interventional data, particularly regarding SCFAs and butyrate supplementation, are derived from preclinical animal models, and extrapolation to humans should be undertaken with caution. Thus, compared with the brain, cardiopulmonary, and hematopoietic axes, the gut-skin axis currently provides the most indirect and speculative evidence for a link with RE. It mainly highlights the broader principle that radiation can disrupt barrier-associated microbiomes and that gut-derived metabolites may influence epithelial repair, rather than establishing skin irradiation as a proven route to RE.

### 5.4 Gut-bone marrow axis

The gut-bone marrow axis positions the intestinal microbiota as a hub whose circulating signals reach the marrow niche, where stromal and immune cells sense and regulate HSPC quiescence, proliferation and lineage decisions. In turn, infection, inflammation, hypoxia, medications and diet remodel gut communities and metabolism to influence marrow homeostasis and emergency hematopoiesis^86^. Through a gut-hematopoietic-skeletal axis, microbiota and their metabolites have been reported to afford radioprotection to marrow and bone. Microbial metabolites bidirectionally tune hematopoiesis. I3A, a tryptophan-derived microbial metabolite, enhances HSPC dormancy, suppresses reactive oxygen species and reduces apoptosis, thereby ameliorating radiation-induced hematopoietic injury^87^. Conversely, a *Bacteroides acidifaciens*-dominant consortium impairs HSC recovery via raffinose metabolism and the bile acid-FXR-NF-κB axis; the FXR inhibitor ursodeoxycholic acid (UDCA) reverses this effect^88^. Dietary fiber and its metabolites are radioprotective. A colon-retentive inulin gel increases beneficial taxa and SCFAs, boosts anti-inflammatory mediators, preserves barrier integrity and protects hematopoiesis^89^. Microbially derived lactate activates LepR^+^ marrow stromal cells to secrete stem cell factor (SCF), promoting hematopoiesis and erythropoiesis; a single oral administration of lactate-producing bacteria yields hematopoietic benefits lasting up to 16 weeks^90^. SCFAs also preserve bone mass and prevent radiation-associated bone loss: propionate and butyrate inhibit osteoclast differentiation and resorption and induce metabolic reprogramming; short-term propionate supplementation reduces bone-resorption markers in healthy humans^91^. However, direct evidence demonstrating that bone marrow irradiation reciprocally drives gut dysbiosis and contributes to RE pathogenesis is still lacking. Therefore, the gut-bone marrow axis in the specific context of RE should currently be regarded as a hypothesis-generating concept.

In summary, building on the well-established roles of the gut microbiota in multi-organ-related diseases through gut-X axes^92^, we propose a potential framework in which radiotherapy, in the context of gut-multi-organ axes, may drive intestinal inflammation by perturbing the gut microbiota. Gut-derived metabolites can further reach distant organs to modulate stem-cell behavior and tissue metabolism, forming a cross-organ network with radioprotective potential. Given the current paucity of direct evidence linking gut-organ axes to RE, this section synthesizes broader literature on radiation-associated injury and the microbiota to provide a conceptual and mechanistic framework that may guide future investigations and inform microbiota-targeted strategies for RE prevention and management (Table 2).

## 6. Microbiome-modulating strategies and clinical translation

### 6.1 Individualized risk stratification and precision prophylaxis

Individualized risk assessment underpins precision prevention of RE. By integrating clinical parameters, microbiome features and multi-omics data, high-risk patients can be identified pre-radiotherapy and targeted preventive strategies deployed.

Baseline gut composition has shown predictive potential. In a prospective cervical-cancer cohort, pre-treatment enterotyping had strong prognostic value: a *Faecalibacterium*-predominant enterotype conferred a 4.4-fold higher risk of severe acute RE^50^, indicating that simple enterotyping can rapidly flag high-risk individuals in practice. In prostate cancer, the MICLIDE decision-tree model, built on six genera (*Faecalibacterium*, *Bacteroides*, *Parabacteroides*, *Alistipes*, *Prevotella* and *Phascolarctobacterium*), effectively stratified gastrointestinal toxicity risk and warrants further prospective validation before clinical implementation^93^. Targeted biomarkers further enhance performance: combined quantification of the bacterium *Erysipelatoclostridium* and its metabolite ptilosteroid A achieved superior prediction (AUC = 0.875), outperforming standalone clinical parameters^94^.

**Figure 6:**
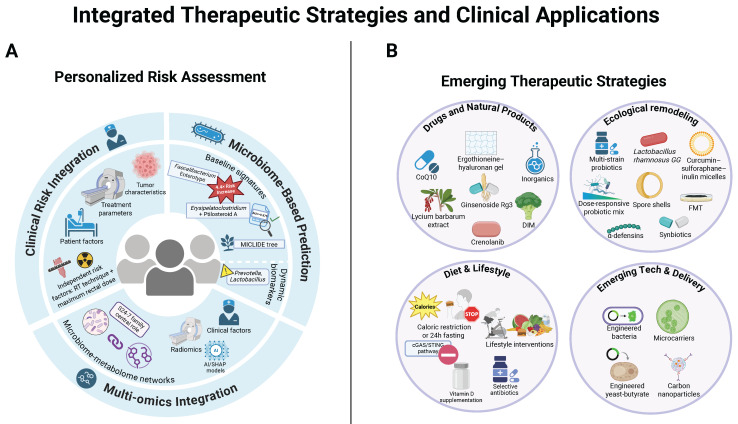
** Integrated therapeutic strategies and clinical applications. (A)** Personalized risk assessment integrates clinical factors, microbiome signatures and multi-omics/AI models to stratify radiotherapy-related outcomes. **(B)** Emerging therapeutic strategies span pharmacologic agents, ecological microbiome remodeling, diet-lifestyle interventions, and advanced delivery platforms targeting radiation-related toxicity. cGAS-STING, cyclic GMP-AMP synthase-stimulator of interferon genes.

Dynamic monitoring adds actionable value. Large-animal studies corroborate dose-response biology, showing linear relationships between radiation dose and the abundance of key genera such as *Prevotella*, *Lactobacillus* and *Elusimicrobium*/*Treponema*^16^,^95^. Multi-omics integration markedly boosts predictive accuracy: microbiome-metabolome analyses not only identify risk-linked taxa but also reveal functional mechanisms, for example the central regulatory role of the S24-7 family within intestinal metabolic networks^95^. Radiomics further refines prognostication: combining radiomics with clinical factors increased AUC from 0.6641 to 0.6855, while interpretable SHAP-based models preserved accuracy and provided transparent attribution^96^. An integrated radiomics nomogram that fuses dose-volume and image-derived features accurately predicts severe radiation proctitis in post-operative cervical-cancer patients, informing individualized planning. Risk-tiered prophylaxis can then be implemented. Low-risk patients generally require standard supportive care. For intermediate-risk patients, evidence-supported probiotics or synbiotic formulations, such as those containing LGG and *Bifidobacterium longum*, may be considered in selected clinical contexts. High-risk patients merit multimodal interventions: prophylactic anti-inflammatory therapy (for example, triamcinolone acetonide 40 mg intramuscularly on days 1, 11 and 21, which reduces gastrointestinal and genitourinary toxicity during pelvic radiotherapy)^97^, intensified nutritional support, combined probiotic-prebiotic therapy and consideration of advanced radiotherapy techniques. Finally, imbalance of the vWF/ADAMTS13 axis has emerged as a potential preventive target^13^ (Figure 6A).

### 6.2 Clinical strategies for microbiome intervention

Although diverse microbiome-modulating strategies have shown intestinal protective effects in experimental models, their clinical translation remains constrained by several factors. First, most interventional evidence still comes from animal studies, whereas randomized controlled trials in patients receiving radiotherapy remain limited. Second, strains, doses, timing and treatment duration differ widely across studies, and no standardized regimen has yet been established. Finally, for patients with cancer, any radioprotective strategy must demonstrate that it does not simultaneously protect tumor cells or compromise the antitumor efficacy of radiotherapy; this issue has not been systematically addressed in many studies. These considerations should guide the interpretation of the intervention studies summarized below.

#### 6.2.1 Ecological remodeling

Multi-strain probiotics outperform single strains in radioprotection. A consortium comprising Bifidobacterium longum BL21, *Lactobacillus paracasei* LC86 and *L. plantarum* Lp90 synergistically mitigates radiation-induced intestinal injury by promoting intestinal stem-cell proliferation and differentiation, strengthening barrier integrity and modulating oxidative stress and inflammation^98^. Orally administered composite probiotics show a clear dose-response in attenuating murine radiation enteropathy; during the post-irradiation critical window they suppress pathogenic taxa such as the *Escherichia-Shigella* cluster and increase taxa generally associated with SCFA production, such as Lachnospiraceae^17^. Spore-shell biomaterials offer a live-microbe-free alternative resilient to gastric acid and irradiation. Spore shells derived from Bacillus species (for example, *Bacillus subtilis* and *B. coagulans*) efficiently alleviate radiation-induced gut injury, improve survival and restore microbial homeostasis in mice^99^. Host defense peptides also contribute: intestinal α-defensins facilitate recovery by reshaping the microbiome and fecal metabolome^100^.

Synbiotics leverage prebiotic-probiotic synergy, but most supporting data currently come from preclinical studies. Clinically, in a randomized controlled trial in prostate cancer, a synbiotic containing *Lactobacillus reuteri* plus dietary fiber significantly alleviated acute radiation proctitis symptoms, especially tenesmus and defecatory urgency during urination, improving quality of life, with peak benefit at weeks 2-3^101^. By contrast, the following findings are derived from mouse models and other preclinical work. Inulin hydrogel combined with multi-strain probiotics significantly improves survival in irradiated mice, outperforming either component alone; as a prebiotic carrier, inulin prolongs probiotic gut residence and cooperatively enhances SCFA production^102^. For prevention of radiation-induced colonic fibrosis, inulin promotes expansion of context-dependent SCFA-producing or fermentative taxa, including Ruminococcaceae, *Bacteroides acidifaciens* and *Clostridium papyrosolvens*, reducing collagen deposition and α-SMA expression^103^. A colon-retentive inulin gel similarly protected both the hematopoietic system and the gastrointestinal tract in irradiated mice, in parallel with increased SCFAs output and elevated anti-inflammatory cytokines IL-22 and IL-10, in a microbiota-dependent manner^89^. In mouse models, konjac glucomannan (KGM), another prebiotic, fosters beneficial taxa and SCFAs and confers protection against both total- and abdominal-irradiation injury^104^. Multifunctional oral curcumin-thione-inulin conjugate micelles integrate direct antioxidant activity (radical scavenging) with inulin-mediated dysbiosis correction, yielding multilevel protection^105^. Together, these data suggest that inulin- and KGM-based prebiotics and synbiotics are promising candidates, but their efficacy and safety in patients receiving radiotherapy remain to be defined.

FMT is being explored as a microbiome-based intervention. FMT ameliorates murine RE by restoring microbiota-mediated tryptophan metabolism, increasing production of I3A and related metabolites to dampen oxidative stress and inflammation^106^. Microbiota harvested from “elite survivor” mice that live through high-dose irradiation provide pronounced radioprotection; Lachnospiraceae and Enterococcaceae were identified as key protective families, with propionate and tryptophan metabolites underpinning durable benefits^21^. Sex-matched FMT significantly improves survival, gastrointestinal function and epithelial integrity after irradiation, and combines favorably with bone-marrow transplantation^107^. Targeted, composition-guided consortia are advancing; inocula enriched for Lachnospiraceae protect ISCs and preserve barrier integrity.

#### 6.2.2 Pharmacological and natural-product interventions

Natural products, including plant-derived compounds and fungus-derived bioactive polysaccharides, have attracted interest as potential protectants against radiation-induced intestinal injury, although most supporting evidence remains preclinical. Clinically, epigallocatechin-3-gallate (EGCG) protects via a novel gut microbiota/D-tagatose/AMPK axis, preserving mucosal architecture and promoting regeneration. In pelvic-cancer patients, 400 mg/day EGCG reduced the incidence of grade ≥2 RE from 65% to 24.3%^108^. By contrast, the following data are derived from rat or mouse models. Ginsenoside Rg3 suppresses the TLR4/MyD88/NF-κB inflammatory cascade while rebalancing the gut microbiota. At a high dose (80 mg/kg), Rg3 achieved efficacy comparable to dexamethasone and exhibited a greater capacity to remodel microbial communities^65^. The cruciferous-vegetable metabolite 3,3′-diindolylmethane (DIM) confers marked protection against TAI-induced gut injury by enhancing survival and function of *Lgr5*^+^ ISCs and progeny, activating the Nrf2 antioxidant pathway to mitigate oxidative stress, and restoring radiation-induced dysbiosis^109^. Lycium barbarum (goji) extract alleviates radiation injury through multi-pathway mechanisms and increases beneficial taxa such as *Turicibacter* and *Akkermansia*^110^. The vanillin-derived small molecule VND3207 precisely tunes the p53/NOXA axis to sustain DNA repair without triggering excessive apoptosis, while correcting dysbiosis; it significantly improved survival after lethal-dose irradiation in mice^111^. Fungus-derived polysaccharides may represent a promising class of natural radioprotective agents, and *Tremella fuciformis* polysaccharide TFP-23 has been reported to mitigate radiation-induced intestinal injury in mice by reducing colon shortening and crypt loss. This protection was associated with partial restoration of radiation-disrupted gut microbiota, including increased microbial diversity, reduced Proteobacteria expansion and enrichment of beneficial taxa such as *Akkermansia*, *Bacteroides*, *Faecalibacterium* and *Oscillospirales*, although further validation is still required^112^.

Conventional drugs also provide benefit. In patients with chronic RE, a pentoxifylline-vitamin E regimen improved symptoms of chronic RE by transcriptionally downregulating TGF-β1, reducing SOMA scores by 41% and 80% at 6 and 18 months, respectively^67^. In rat models, coenzyme Q10 attenuates inflammation and fibrosis by inhibiting NF-κB/TGF-β/MMP-9 signaling and increases the relative abundance of Bacteroidetes, *Akkermansia* and SCFA producers^64^. In mouse models, inorganic agents have also been reported to afford additional protection. Selenium nanoparticles (SeNPs) ameliorate radiation colitis by modulating the cGAS-STING pathway, suppressing X-ray-induced excess ROS and DNA damage, and reshaping the inflammatory immune microenvironment^113^. Similarly, in mouse models, dietary nitrate prevents whole-body-irradiation-induced colonic injury by activating the NO3-/NO2-/NO pathway, limiting oxidative stress and maintaining microbiome homeostasis^114^.

Epigenetic and cell-death pathway modulators offer complementary strategies, but current mechanistic evidence comes predominantly from animal models. The histone deacetylase inhibitor trichostatin A (TSA) mitigates gastrointestinal toxicity via epigenetic and anti-inflammatory effects and shifts the microbiome by increasing Verrucomicrobia and *Lactobacillus*^115^. Ferrostatin-1, a specific ferroptosis inhibitor, suppresses both apoptosis and ferroptosis after irradiation, through inhibition of p53-mediated apoptotic signaling and restoration of microbial balance, thereby improving survival^7^. The HSP90 inhibitor NVP-AUY922 downregulates epoxide hydrolase 1 (EPHX1), curbing radiation-induced intestinal inflammation and remodeling gut communities^116^. The PDGF-C signaling pathway is a key regulator of radiation proctopathy; its inhibitor crenolanib attenuates colorectal tissue injury by blocking the PDGF-C/PDGFRs/ETV1/CXCR4 axis^11^.

Other notable interventions include an ergothioneine-hyaluronic acid gel, which reduces apoptosis and inflammation while improving microbial balance^117^; in rat models, combined supplementation with β-hydroxy-β-methylbutyrate (HMB), L-glutamine and L-arginine to promote protein synthesis, suppress proteolysis and reinforce epithelial structure and function^63^; and in mouse models, hydrogen-rich water, which exerts antioxidant and anti-inflammatory effects via MyD88 signaling while stabilizing the gut microbiota^118^.

#### 6.2.3 Diet and lifestyle interventions

Dietary and lifestyle measures provide non-invasive, readily implementable strategies for mitigating radiation-induced intestinal injury. Exercise is a simple, effective lever: low-intensity walking (1 m per minute, 35 minutes daily) increased the relative abundance of *A. muciniphila* and attenuated radiation enteropathy without compromising tumor control^119^.

Dietary restriction, either 30% caloric restriction or a 24-hour fast, ameliorates acute intestinal injury by suppressing cGAS/STING activation^120^. These strategies exhibit sex-dependent protective effects: 30% caloric restriction in females primarily reduces pro-inflammatory taxa (for example, *Helicobacter* and Desulfovibrionaceae), whereas in males it enriches SCFA producers (for example, *Faecalibaculum* and *Lactobacillus*), thereby improving barrier function^121^. By contrast, dietary methionine supplementation exacerbates ionizing-radiation-induced gastrointestinal syndrome, accelerating the expansion of pathogenic Burkholderiales and thus warrants caution^122^. High-fiber intake increases *Bacteroides acidifaciens* and can significantly enhance tumor radiosensitivity^123^. Fiber supplementation can both potentiate tumor response and mitigate gut toxicity: psyllium plus resistant starch delivered superior radiosensitization, whereas psyllium plus inulin afforded better protection against acute enterotoxicity^124^.

Circadian alignment also shapes radiosensitivity. Disruption of circadian rhythms induces dysbiosis and weakens radiotolerance, whereas mice maintained under a standard 12-hour dark/12-hour light cycle display greater resistance to radiation injury^125^. Vitamin D supplementation protects by preserving barrier integrity and modulating microbiota composition^126^. Selective antibiotic strategies (for example, vancomycin) that deplete butyrate-producing bacteria may, in certain settings, both reduce intestinal toxicity and enhance the anti-tumor efficacy of radiotherapy^127^.

#### 6.2.4 Emerging technologies and delivery systems

Engineered microbes are being investigated for both radiosensitization and radioprotection. A tumor-targeting probiotic, *Escherichia coli* Nissle 1917, markedly increases catalase secretion within the tumor microenvironment (TME), catalyzing tumor-derived H_2_O_2_ into O_2_. By relieving hypoxia and improving oxygen availability, this approach enhances radiation-induced ROS generation and achieves radiosensitization in mice^128^. An IL-22-secreting engineered probiotic promotes direct intestinal epithelial repair and restores bone-marrow hematopoiesis; notably, administration as late as 24 hours post-irradiation significantly improves survival, offering a practical window for delayed intervention^129^. A butyrate-engineered yeast releases butyrate to restore GPR109A expression, protecting the intestine and, via the gut-heart axis, mitigating radiation-induced cardiac injury while activating the cardioprotective genes Nppa and Sgcg, illustrating the multi-organ protective potential of engineered strains^130^.

Targeted delivery systems can substantially amplify microbial and metabolic therapies. An oral micro-carrier based on the microalga Spirulina efficiently delivers amifostine (AMF), protecting the small intestine from radiation injury and stabilizing the gut microbiota, particularly by increasing beneficial taxa such as *Lactobacillus*, *Prevotella* and *Alloprevotella*, thereby providing broad radioprotection^131^. A metal-organic framework (MOF) platform constructed from zinc ions and 2-methylimidazole provides pH-responsive, intestinal release of sitagliptin alongside Zn2+, affording multifaceted protection through a microbiota-SCFAs-tight junction axis^132^. Clinically approved carbon nanoparticles (CNSI), administered orally, leverage potent free-radical scavenging and intestinal targeting to preserve crypt stem cells and maintain microbial balance, with notable enrichment of probiotic *Lactobacillus* and the order Bifidobacteriales^133^.

From a radiation-physics perspective, modern ultra-high-dose-rate (FLASH) radiotherapy, by virtue of its unique temporal pulse structure, spares normal tissues. Implemented as a single pulse with a mean dose rate ≥ 280 Gy/s, FLASH significantly attenuates damage to the intestinal microbiota and mucosal tissues^134^, providing a complementary physical modality to protect the gut ecosystem (Figure 6B).

### 6.3 Clinical implementation of integrated therapeutic strategies

Management of RE should be tailored to disease phase. In the acute phase (during radiotherapy to within 3 months after completion), priorities are diarrhea control and preservation of epithelial barrier integrity. In the chronic phase (beyond 3 months), the focus shifts to antifibrotic measures and quality-of-life optimization. Interventions such as nicotinamide mononucleotide (NMN) and inulin modulate the gut microbiota—particularly increasing Bacteroidetes, *Akkermansia* and short-chain fatty-acid producers—and thereby indirectly mitigate fibrogenesis^68^,^103^. For severe cases refractory to conservative therapy, anatomically precise resection of diseased bowel remains an effective salvage option; in one series, no recurrence was observed over a median 42-month follow-up^66^.

From a translational perspective, clinical confounders remain a major challenge. Although clinical studies have controlled for antibiotic use during fecal sample collection, many patients with locally advanced or metastatic cancer receive cytotoxic chemotherapy and/or undergo surgical resection, both of which can substantially perturb intestinal ecology, mucosal immunity, intestinal anatomy and microbial niches. These factors may interact with radiation-induced epithelial injury and dysbiosis, thereby complicating causal attribution of microbiome alterations to radiotherapy or RE alone. In addition, inter-individual variability related to age, sex, diet, geography, lifestyle, and host genetic background can influence baseline microbiome composition and function and may partly explain the heterogeneity of microbial signatures and clinical RE phenotypes observed across cohorts. Therefore, such heterogeneity should be interpreted in a balanced manner, with explicit consideration of how clinical management and patient-level factors may modulate both the microbiota and the risk of RE. Taken together, these clinical constraints underscore the gap between controlled research settings and everyday oncology practice and must be carefully considered when designing and interpreting microbiome-based interventions.

In parallel with these clinical constraints, much of the available evidence still comes from preclinical studies, particularly mouse models, and should therefore be regarded primarily as mechanistic and conceptual insight rather than definitive clinical proof. Nevertheless, these studies collectively highlight that host factors, including age, sex, genetic background, metabolic state/comorbidities and circadian status, substantially influence the onset and trajectory of RE. Risk-assessment frameworks are coalescing from clinical phenotyping to microbiome readouts and multi-omics integration. Microbiome modulation is evolving from conventional probiotics to precision, engineered live biotherapeutics. Layered, multi-target strategies offer promising avenues for the prevention and treatment of RE. As microbiome science and enabling technologies continue to advance, RE management may become increasingly precise and effective, with meaningful improvements in patient outcomes and quality of life. However, despite substantial mechanistic progress in animal studies, interspecies differences in host-microbiome composition, metabolism and treatment response mean that not all preclinical findings will be reproducible in humans. Accordingly, these strategies should be interpreted with caution and will require rigorous, well-designed clinical trials before they can be translated into routine practice.

## 7. Conclusions and perspectives

Research on RE is moving toward multidimensional integration, with three priorities emerging. First, multi-omics integration will deepen understanding of host-microbiome crosstalk^95^. In this context, future studies should integrate bacterial, fungal, and viral microbiome data to better define the host-microbiome interaction network in RE. Prospective clinical studies are needed to validate microbiome biomarkers^135^. Second, in translational medicine, individualized risk-assessment models based on radiomics and artificial intelligence are advancing; radioprotective microbes and metabolites identified from radiation survivors provide actionable targets for next-generation protectants^21^. Third, on the clinical front, systems-level regulation along the gut-multi-organ axis, together with rigorous assessment of the long-term efficacy and safety of microbial interventions, warrants in-depth investigation. Clinical practice should adopt systematic, personalized strategies: perform pre-radiotherapy microbiome profiling to identify high-risk patients; implement tumor-specific management and stepwise regimens tailored to the acute and chronic phases; deploy evidence-based microbial interventions; and integrate nutritional support that recognizes the microbiome as a key transducer of host nutrient signaling^136^. Multidisciplinary teamwork is essential to optimize outcomes.

Overall, RE has evolved from a concept of purely local inflammation to a systemic disease involving complex cell-death programs, oxidative stress, immune dysregulation and microbiome imbalance. Its clinical manifestations exhibit clear dependence on tumor type, anatomical site and host factors. The gut microbiome appears to contribute to the initiation, progression and resolution of RE, with effects extending from the intestinal compartment to multi-system involvement^8^. Dynamic equilibria between the microbiome and specific metabolites, including SCFAs, bile acids and tryptophan derivatives, provide critical radioprotective targets. Diagnostics and risk assessment are shifting from single-parameter approaches to personalized, multi-omics models, while therapy is moving from symptomatic care to mechanism-based, multi-target interventions. In the era of precision medicine, management is converging on a “predictive, preventive, personalized and participatory” continuum; integrating microbiome-modulating, individualized strategies will continue to drive clinical innovation, with the goal of delivering precise protection and optimal prognosis for every patient.

## Figures and Tables

**Table 1:** Gut bacteria and microbial metabolites involved in RE progression

Bacteria	Metabolite	Effect	Mechanism	Model	Refs.
*Akkermansia muciniphila*	Propionate	Protective (barrier)	Strengthens epithelial barrier (ZO-1, occludin and MUC2) via GPR43 activation.	Murine models; pelvic RT patient cohorts.	20, 32
*Akkermansia muciniphila*	3-Hydroxybutyrate (3HB)	Protective (anti-inflammatory)	Blocks GPR43-mediated IL-6 signaling in rectal mucosa, lowering GPR43 and IL-6 expression.	Human cohorts; murine models.	34
*Faecalibaculum rodentium*	Butyrate	Protective (intestinal & hematopoietic)	Prevents ERK-dependent PKM2 nuclear translocation and reduces p53/BAX-mediated apoptosis in HSPCs.	Murine models.	33
*Faecalibacterium prausnitzii*	-	Radioprotective	Maintains the Sox-9⁺ stem/progenitor pool, stimulates crypt epithelial proliferation.	Rat models (male Sprague-Dawley rats).	36
-	Indole-3-propionic acid (IPA)	Protective (systemic)	Gut microbiota-derived IPA activates PXR-ACBP axis and enhances antioxidant and metabolic homeostasis.	Murine models.	21, 47
-	Indole-3-aldehyde (I3A)	Protective (barrier & regeneration)	Activates AhR/IL-10/Wnt signaling to promote epithelial proliferation and ISC-mediated regeneration.	Murine models.	41
*Peptostreptococcus*	Indoleacrylic acid (IA)	Protective (anti-inflammatory, barrier)	Activates Nrf2-ARE signaling and upregulates antioxidant and barrier-related genes.	*In vitro* systems and human cohorts.	49
-	Urolithin A (UroA)	Protective (gut), Sensitizing (tumor)	Inhibits p53-mediated apoptosis, remodels gut microbiota and reduces tumor-associated macrophages.	Murine models.	48
-	Secondary bile acids / bile extract	Protective (ISC-driven regeneration)	Activates epithelial TGR5-YAP1 signaling to promote ISC proliferation and mucosal regeneration.	Murine models.	44, 45
-	Lactate	Protective (epithelial development)	Engages GPR81 on Paneth and stromal cells to induce Wnt3 and drive Lgr5⁺ ISC-mediated regeneration.	Murine models.	46
-	H₂S	Protective (antioxidant)	Activates the Keap1/Nrf2/ARE cytoprotective axis to enhance antioxidant defenses.	*In vitro* systems.	49
*Weissella cibaria*	Dextran and propionate	Protective (mucosal barrier)	Dextran increases propionate, reshapes gut microbiota and strengthens the mucus layer and tight junctions.	Murine models.	35
*Lactobacillus rhamnosus*	Lipoteichoic acid (LTA)	Protective (crypt/ISC protection)	Activates TLR2-MyD88 signaling and induces CXCL12 to recruit COX-2⁺ mesenchymal stem cells.	Murine models.	37, 38, 51

**Table 2:** Microbiota- and metabolite-mediated gut-organ axes implicated in radiation-induced injury and protection

Axis	Microbiota / metabolite / intervention	Change / role in radiotherapy context	Radiation-related outcome (distant organ)	Key mechanisms / pathways (keywords)	Model	Refs.
Gut-brain	miR-34a-5p / BDNF axis	miR-34a-5p increase; hippocampal BDNF decrease	Cognitive impairment, memory deficits	Microbiota-dependent regulation of miR-34a-5p and neurotrophic signaling	Murine models.	72
	*Parabacteroides*, *Sutterella*, *Desulfovibrio*	Increased after pelvic irradiation	Neuronal death, reduced neuronal maturity, altered exploratory behavior	Gut-brain signaling, astrocyte activation, reduced hippocampal plasticity markers	Murine models.	73
	*Muribaculum*, *Parabacteroides*	Increased at early timepoints after focal cranial irradiation	Associated with changes in behavior and metabolism	Reshaped gut community, altered arginine and proline metabolism	Murine models.	74
	*Akkermansia*, *Helicobacter*	Decreased at later timepoints after cranial irradiation	Linked to sustained metabolic and neural alterations	Fecal, serum and cortical metabolites, including tryptophan pathway	Murine models.	74
	Eleutheroside E	Oral pretreatment before irradiation	Attenuates radiation-induced cognitive dysfunction	*Lactobacillus* increase, *Helicobacter* reduction, PKA/CREB/BDNF activation	Murine models.	75
	*Lactobacillus* microcapsules	Probiotic supplementation after irradiation	Improves hippocampal pathology, reduces anxiety-like behavior, enhances memory	Enriches Lachnospiraceae, *Blautia*, *Lactobacillus*; lowers inflammatory cytokines; improves intestinal milieu	Murine models.	76
Gut-cardio pulmonary	L-histidine / imidazole propionate (ImP)	Oral L-histidine; microbial conversion to ImP	Protects heart and lung from structural injury and collagen accumulation.	*Akkermansia* and *Lactobacillus* increase, ImP inhibit the pyroptotic lung cell death	Murine models.	82
	Gut microbiota-derived prostaglandin F2α (PGF2α)	Chest irradiation remodels gut microbiota and promotes circulating PGF2α signaling	Mitigates radiation-induced lung injury	PGF2α crosses the intestinal barrier and activates FP/MAPK/NF-κB signaling	Murine models.	79
Gut-skin	“Type C” skin microbiome	Characteristic pattern in a subset of RID patients	Predicts chronic or delayed-healing RID, especially in diabetes	Pathobiont overgrowth and persistent local inflammation	Human cohorts.	84
	SCFAs, especially butyrate	Reduced with low-fiber diet; restored by dietary fiber or supplementation	Improved skin-barrier integrity; potential mitigation of RID	Butyrate enhances mitochondrial β-oxidation and keratinocyte differentiation	Murine models.	85
Gut-bone marrow	Indole-3-aldehyde	Tryptophan-derived microbial metabolite	Ameliorates radiation-induced hematopoietic injury	Enhances HSPC dormancy, reduces reactive oxygen species and apoptosis	Murine models.	87
	Bacteroides acidifaciens-dominant consortium	Overrepresentation after specific microbial colonization	Impairs HSC recovery after irradiation	Activates bile acid-FXR-NF-κB axis, damages HSC niche	Human cohorts; murine models.	88
	Ursodeoxycholic acid (UDCA)	FXR inhibitor given after irradiation	Reverses consortium-induced HSC impairment	Blocks FXR-NF-κB signaling; restores hematopoietic recovery	Human cohorts; murine models.	88
	Colon-retentive inulin gel	Fermentable fiber targeted to the colon	Protects hematopoiesis and intestinal barrier after irradiation	Increases beneficial taxa and SCFAs and boosts anti-inflammatory mediators	Murine models.	89
	Lactate-producing bacteria	Single oral administration; increased systemic lactate	Long-lasting enhancement of hematopoiesis and erythropoiesis	Activates LepR-positive marrow stromal cells to secrete stem cell factor (SCF)	Murine models.	90
	Propionate and butyrate (SCFAs)	Supplementation or high-fiber diets	Preserve bone mass; prevent radiation-associated bone loss	Inhibits osteoclast differentiation, induces metabolic reprogramming and lowers bone resorption markers	Human cohorts; murine models.	91
